# G-Protein/β-Arrestin-Linked Fluctuating Network of G-Protein-Coupled Receptors for Predicting Drug Efficacy and Bias Using Short-Term Molecular Dynamics Simulation

**DOI:** 10.1371/journal.pone.0155816

**Published:** 2016-05-17

**Authors:** Osamu Ichikawa, Kazushi Fujimoto, Atsushi Yamada, Susumu Okazaki, Kazuto Yamazaki

**Affiliations:** 1 Genomic Science Laboratories, Sumitomo Dainippon Pharma. Co. Ltd, Osaka, Osaka, Japan; 2 Department of Applied Chemistry, Nagoya University, Nagoya, Aichi, Japan; Indian Institute of Technology Kanpur, INDIA

## Abstract

The efficacy and bias of signal transduction induced by a drug at a target protein are closely associated with the benefits and side effects of the drug. In particular, partial agonist activity and G-protein/β-arrestin-biased agonist activity for the G-protein-coupled receptor (GPCR) family, the family with the most target proteins of launched drugs, are key issues in drug discovery. However, designing GPCR drugs with appropriate efficacy and bias is challenging because the dynamic mechanism of signal transduction induced by ligand—receptor interactions is complicated. Here, we identified the G-protein/β-arrestin-linked fluctuating network, which initiates large-scale conformational changes, using sub-microsecond molecular dynamics (MD) simulations of the β_2_-adrenergic receptor (β_2_AR) with a diverse collection of ligands and correlation analysis of their G protein/β-arrestin efficacy. The G-protein-linked fluctuating network extends from the ligand-binding site to the G-protein-binding site through the connector region, and the β-arrestin-linked fluctuating network consists of the NPxxY motif and adjacent regions. We confirmed that the averaged values of fluctuation in the fluctuating network detected are good quantitative indexes for explaining G protein/β-arrestin efficacy. These results indicate that short-term MD simulation is a practical method to predict the efficacy and bias of any compound for GPCRs.

## Introduction

G-protein-coupled receptors (GPCRs), which constitute one of the largest families of membrane-bound receptors, are encoded by more than 800 genes in the human genome [1], and more than 25% of available drugs target GPCRs [2,3]. Binding of these drugs results in the induction or inhibition of signal transduction mediated by cytoplasmic effector proteins such as G proteins and β-arrestins. The signal transduction induced by various ligands is mainly characterized by the strength of signaling and the bias of signaling in the G protein and β-arrestin pathways. Each GPCR ligand has a different strength of signaling, which is commonly referred to as efficacy, and the ligands are classified according to their efficacies, for example, full agonists, partial agonists, neutral antagonists, and inverse agonists [4,5]. These differences in efficacy significantly affect the clinical properties of GPCR ligands. For drugs that target the β_2_-adrenergic receptor (β_2_AR), full agonists offer therapeutic advantages over partial agonists in acute severe asthma, although full agonists can potentially cause more adverse effects [6]. On the other hand, a number of GPCR ligands, including the US Food and Drug Administration-approved β-blockers [7,8], elicit different degrees of signaling in the G protein and β-arrestin pathways, which is called “functional selectivity” or “biased signaling” [9]. These differences in biased signaling are also thought to affect the clinical properties. Therefore, controlling both efficacy and bias in signal transduction is considered crucial in designing drugs that are more effective and safer.

Structural analyses of GPCRs have clarified the multiple conformations of various ligand-bound receptors, representing fundamental knowledge for understanding the mechanism of ligand efficacy and bias. Crystal structures have been determined for a number of GPCRs [10,11], and they share a similar global conformation [12,13]. The crystal structures of β_2_AR, which is an archetypal GPCR, are generally classified into two conformation types [13]. The first is typified by β_2_AR complexed with the inverse agonist carazolol [Protein Data Bank (PDB) ID: 2RH1] [14], which represents a snapshot of the inactive state (**R**). The second is represented by β_2_AR with a full agonist, BI-167107, and a G protein (PDB ID: 3SN6) [15], which likely represents a snapshot of the G-protein-active state (R*). In another agonist-bound β_2_AR structure without an intracellular binding partner (PDB ID: 3PDS) [16], β_2_AR is almost identical to the inverse-agonist-bound β_2_AR. These crystal structures suggest that, even though agonist binding likely increases the population of active states, most of the receptor remains in the **R** state in the absence of a G protein. Comparison of the structures of the **R** and R* states shows small changes in the tertiary contacts of the seven transmembrane helices, small movements within the ligand binding site, and more profound outward movement of helix 6 on the intracellular surface (14 Å difference at the C_α_ carbon of Glu268^6.30^), which enable the G protein to bind the intracellular surface of the receptor [15,17]. On the other hand, complementary information has been lacking for the β-arrestin-active state (R**), although a low-resolution model for the overall conformation of the β_2_AR-β-arrestin-1 complex has been visualized using electron microscopy [18].

On the basis of these snapshots of the multi-states, the dynamics of β_2_AR has been analyzed using NMR probe studies with the chemical ^19^F-labeling of cysteines or isotopic labeling of ^13^C_ε_H_3_-methionines [19–21]. These studies have revealed that the conformational states exchange on a microsecond to millisecond time scale and that each of these states is the ensemble of sub-nanosecond-lived substrates. In addition, the population and amplitude of motion of the given states are modulated by agonists and inverse agonists. For biased signaling, differences in the population of conformational states have been attributed to the differences between helix perturbations for G-protein- and β-arrestin-biased ligands [22–24]. Molecular dynamics (MD) simulation is a useful method for calculating time-dependent change and dynamics at an atomic resolution, especially for analyzing the pathway of conformational change and dynamics from femtoseconds to milliseconds. In a previous study, all-atom MD simulations for a total of more than 650 μs were performed using a specialized supercomputer (Anton), revealing the pathway for conformational change [25]. This study clarified the transition pathway starting from the R* state into the **R** state upon removal of the G protein or its mimetic nanobody. These experimental and computational studies have elucidated the main mechanisms underlying GPCR conformational change.

In order to discover drugs that provide finer control of GPCR signaling, there is a strong need for a practical method to predict the efficacy and bias of any compound [26]. The above-mentioned MD simulation of the pathway offers a possible solution and produces accurate transition pictures; however, performing simulations of the order of tens of microseconds has substantial calculation costs, which becomes problematic when screening many candidate compounds during drug discovery research. In this study, we proposed a practical method for predicting possible drug candidates based on sub-microsecond MD simulations. Our method focuses not on the long-term transition pathway between R* and **R** but on the short-term fluctuation difference between them, which would initiate the large-scale conformational changes. We identified this predominant fluctuation, which is called the efficacy-linked fluctuating network and is composed of “the fluctuating atoms” and “atom–atom couplings”, by performing MD simulations of both the R* and **R** states of β_2_AR with diverse ligands and analyzing the correlation between the sampled dynamics and the G-protein/β-arrestin efficacies.

## Methods

### Dataset of β_2_AR ligands

Fourteen diverse β_2_AR ligands, whose experimental values for G protein and/or β-arrestin efficacy had already been determined, were selected for the efficacy-linked fluctuating network analysis using the following procedure. At first, we choose 11 ligands that were reported in the article by Rajagopal et al. [27]. Their G protein and β-arrestin efficacies were determined under common experimental conditions using the GloSensor assay and the Tango assay, respectively. In order to ensure a diverse range of ligand efficacies, we added two inverse agonists, carazolol and ICI-119551 [18,23,28,29], and one full agonist, BI-167107 [18,30] (Table 1 and S1 Fig). In the efficacy-linked fluctuating network analysis, we simply used the E_max_ value, the maximum signaling effect, as the experimental value of efficacy because the ligands kept binding to the binding pocket of their receptor during our MD simulation. When the experimental values of E_max_ are more than 100%, these values were treated as 100% in this study. We assumed the E_max_ values of the inverse agonists carazolol and ICI-118551 to be 0% and that of the full agonist BI-167107 to be 100% for both G protein and β-arrestin efficacy [22,23,28].

**Table 1:** Summary of the efficacy of 14 β_2_AR ligands.

Abbreviated name	Name	G protein efficacy (E_max_)	β-arrestin efficacy (E_max_)	References
BI	BI-167107	100^a^	100^a^	[18,30]
ISO	Isoprenaline	99.14	80.47	[27]
FEN	Fenoterol	102.2	70.17	[27]
FOR	Formoterol	104.6	99.75	[27]
SAM	Salmeterol	111.8	23.26	[27]
CLE	Clenbuterol	112.8	21.35	[27]
SAL	Salbutamol	107.3	32.41	[27]
NOR	Norepinephrine	107.4	24.89	[27]
DOB	Dobutamine	96.77	4.803	[27]
EPI	Epinephrine	93.52	62.84	[27]
DCI	Dichloroisoproterenol	43.35	2.626	[27]
PIN	Pindolol	15.48	1.979	[27]
CAU	Carazolol	0^b^	0^b^	[23,28]
ICI	ICI-118551	0^b^	0^b^	[18,29]

^a^ We assumed the E_max_ value of the full agonist BI-167107 to be 100% for both G protein and β-arrestin efficacy.

^b^ We assumed the E_max_ values of the inverse agonists Carazolol and ICI-118551 to be 0% for both G protein and β-arrestin efficacy.

We used the same enantiomer of the ligand as in the crystal structure if the crystal structures of the ligand-bound β_2_AR/β_1_AR had been determined, which was the case for *R*-BI-167107 (PDB ID: 3SN6), *S*-carazolol (2RH1), *S*,*S*-ICI-118551 (3NY8), *R*-isoprenaline (2Y03), *R*-salbutamol (2Y04), and *R*-dobutamine (2Y00) (S1 Fig). We chose the *R*-enantiomers of salmeterol, celenbuterol, norepinephrine, epinephrine, and dichloroisoproterenol according to the similarity of the chemical structure to isoprenaline and salbutamol, and the previous report that shows the pharmacological activity of *R*-enantiomers are considerably higher than those of S-enantiomers [31]. We used the *S*-enantiomers of pindolol according to the similarity of the chemical structure to carazolol and ICI-118551, and the previous experimental results [32]. As for fenoterol and formoterol, these are racemic mixtures of *R*,*R*- and *S*,*S*-enantiomers. We selected the *R*,*R*-enantiomers according to the previous studies that show *R*,*R*-enantiomer are the only active isoforms in receptor binding and pharmacological assays [33–35].

### Construction of the simulated systems

The G-protein-active state (R*) of β_2_AR for simulations was prepared using atomic coordinates taken from the β_2_AR–BI-167107–G-protein complex crystal structure (PDB ID: 3SN6) [15], with G protein and the Nb35 nanobody removed. The inactive state (**R**) of β_2_AR for simulations was prepared using atomic coordinates taken from the β_2_AR—carazolol complex crystal structure (PDB ID: 2RH1) [14]. N- and C-terminal residues that were deleted from the crystallized constructs or not resolved in the crystal structures were also omitted from the simulations, resulting in Asp29–Leu342 for the inactive simulations and Glu30–Cys341 for the active simulations. We omitted T4 lysozyme from all simulations and did not attempt to model the unresolved parts of ICL3, as described previously [25]. The corresponding gaps in the ICL3 regions are Asn231–Leu262 for inactive simulations and Glu240–Lys264 for active simulations. All amino acids except for Asp79^2.50^ and Glu122^3.41^ were protonated according to their p*K*_a_ value at neutral pH. Glu122^3.41^, which faces the lipid bilayer, was neutral in all simulations. Asp79^2.50^ was neutral in all simulations, according to the recent long-timescale simulations that showed that the protonation state had little effect on the transition pathway [25]. Chain termini were capped with neutral groups (acetyl and methylamine). All of the crystal waters within 5 Å from the receptor were retained, and other internal water molecules were added with Dowser [36].

For the ligand-bound receptors, all ligands were simulated in the protonated state as described previously [25,37]. We used the same conformation and orientation of the ligand as in the crystal structure if the crystal structures of the ligand-bound β_2_AR/β_1_AR had been determined, which was the case for BI-167107 (PDB ID: 3SN6), carazolol (2RH1), ICI-118551 (3NY8), isoprenaline (2Y03), salbutamol (2Y04), and dobutamine (2Y00). Otherwise, we built ligand-bound receptors by docking the ligands to active and inactive β_2_AR crystal structures using the same protocol as in a previous study [38]. That is, the binding poses of the ligand were developed using a grid-based MD docking algorithm, CDOCKER [39]. In the docking procedure, 10 diverse conformers for the ligand were generated by 1000 steps of MD calculated at 1000 K, and they were then randomly arranged as 10 different poses per conformer into the active site of the receptor. Each of the 100 initial poses was locally optimized using a grid-based simulated annealing and was refined by 250 steps of short energy minimization. These binding poses were then clustered on the basis of a heavy atom Root Mean Square Deviation (RMSD) approach using a 1.5 Å tolerance. Finally, the binding poses with the lowest energy and the distance of Asp113^3.32^ to the protonated nitrogen of the ligand were adapted as the initial poses (S2 Fig).

The orientations of the prepared structures in the membrane were adjusted according to the Orientations of Proteins in Membranes database, which determines the orientation by minimizing the protein transfer energy with respect to angle variables [40]. The 1-palmitoyl-2-oleoyl-sn-glycero-3-phosphocholine (POPC) bilayer of 256 molecules was a pre-equilibrated and fully hydrated around GPCR structure [38,41]. The pre-equilibrated membrane was able to accommodate either the **R** or R* state of β_2_AR with only relatively mild clashes, and they were further relaxed in MD simulations longer than 5 ns. The systems of the active/inactive simulations initially measured roughly 9000 Å^2^ in the x-y-plain (which is the membrane plane) and 120 Å in the z-axis, with about 25,000 water molecules (25,086 for **R** and 25,080 for R*), 75 chloride ions, and 70 sodium ions, accounting for a total of approximately 114,000 atoms.

### MD simulations

The CHARMM22 [42] with CMAP terms [43] was used for the protein, CHARMM36 for the POPC lipids [44], and the CHARMM version of TIP3P model [45] for the water molecules. The force field parameters of the ligands were obtained from the SwissParam server [46]. A cutoff distance of 12 Å was used for the van der Waals and short-range electrostatic interactions, and the long-range electrostatic interactions were computed using the particle mesh Ewald method (PME).

Prior to MD simulations, the ligand–β_2_AR complex was energy minimized using 2000 steps of steepest descent and then equilibrated by 1-ns simulation under NVT conditions at 310 K with a Nosé-Hoover thermostat [47,48]. The NVT simulation was carried out in three separate rounds to allow the position restraints to be gradually released. The first round of NVT was carried out for more than 200 ps with 500 kcal/mol/A^2^ position restraints applied to the ligand–β_2_AR complex. The second round was performed for 400 ps with 10 kcal/mol/A^2^ position restraints applied to the ligand–β_2_AR complex. The third round was performed for 400 ps with 0.1 kcal/mol/A^2^ position restraints applied to the ligand–β_2_AR complex. Covalent bonds between hydrogen and heavy atoms were constrained to their equilibrium values using SHAKE/ROLL and RATTLE/ROLL algorithms [49], and the integration time step was set to 2 fs. The screening parameter, *α*, and the grid number of PME were 3.75×10^-9^ m^-1^ and 128×128×128, respectively. Both minimization and NVT simulations were executed using our originally developed MD software package MODYLAS [50]. After NVT, all the position restraints were removed, and a total of 150 ns of NPT was carried out. The temperature of the systems was maintained at 310 K using Nosé-Hoover thermostat [47,48] and the pressure semi-isotropically maintained at 1 atm pressure using Parrinello-Rahman barostat [51]. All bonds were constrained with the LINCS algorithm [52], and the time integration step was set to 2 fs. Grid space for PME was set to be 1.6 Å, and a fourth-order B-spline was used for interpolation. All NPT simulations were run in GROMACS v4.5.5 [53,54], and trajectory snapshots were saved every 100 ps. For ligand-bound receptors, we performed 150-ns simulations; the first 20 ns was for equilibration, and the next 130 ns of production data was used for later analyses. For the apo receptor, we performed 1-μs simulations; the first 20 ns was for equilibration, and the next 0.98 μs of production data was used for analysis.

### G-protein/β-arrestin-linked fluctuating network analysis

**Fig 1:**
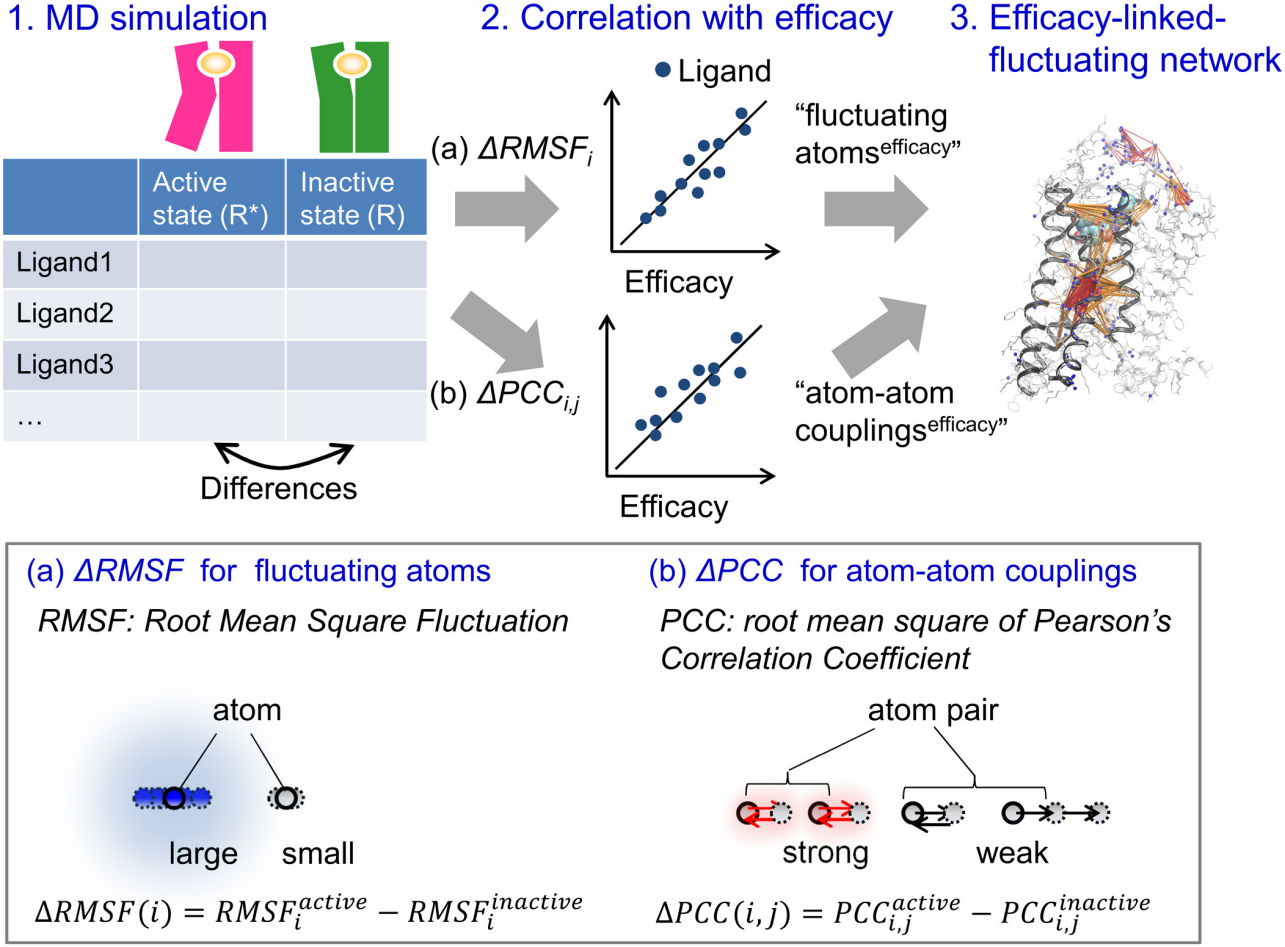
Schematic of the efficacy-linked fluctuating network analysis.

We identified the G-protein/β-arrestin-linked fluctuating network based on the trajectory of short-term MD simulations. This fluctuating network analysis was carried out using two types of fluctuating indexes. One was the *Root Mean Square Fluctuation* (RMSF), which is generally used for quantifying the fluctuation of each atom. The other is the *root mean square of Pearson’s Correlation Coefficient*, and we refer to this root mean square value simply as PCC, which is used for quantifying the cross-correlated fluctuation of each atom–atom pair (Fig 1).

RMSF is defined as follows. Let a protein molecule consist of *N* atoms. At time t, the conformation *r*^(*t*)^ of the protein can be described by a 3*N*-dimensional vector,

$$r^{\left(t\right)}=\left(r_{1}^{\left(t\right)},\ldots ,r_{N}^{\left(t\right)}\right)=\left(x_{1}^{\left(t\right)},y_{1}^{\left(t\right)},z_{1}^{\left(t\right)},\ldots ,x_{N}^{\left(t\right)},y_{N}^{\left(t\right)},z_{N}^{\left(t\right)}\right)$$

A 3*N*-dimentional fluctuation vector of atom i at time t $\;\Delta r_{i}^{\left(t\right)}$ is defined as $\Delta r_{i}^{\left(t\right)}=\left(\Delta x_{i}^{\left(t\right)},\Delta y_{i}^{\left(t\right)},\Delta z_{i}^{\left(t\right)}\right)=r_{i}^{\left(t\right)}-\langle r_{i}\rangle$ using 〈·〉 to denote the ensemble average.

Then, the RMSF of atom i is described as

$$RMSF_{i}=\sqrt{\langle {\left(\Delta x_{i}\right)}^{2}\rangle +\langle {\left(\Delta y_{i}\right)}^{2}\rangle +\langle {\left(\Delta z_{i}\right)}^{2}\rangle }$$ (1)

On the other hand, the PCC was calculated as follows. The 3*N* × 3*N* covariance matrix may be viewed as *N* × *N* submatrices of size 3 × 3. The covariance submatrix between atom i and j, *C*_*ij*_, is described as

$$C_{ij}=\left[\begin{array}{ccc} \langle \left(\Delta x_{i}\Delta x_{j}\right)\rangle & \langle \left(\Delta x_{i}\Delta y_{j}\right)\rangle & \langle \left(\Delta x_{i}\Delta z_{j}\right)\rangle \\ \langle \left(\Delta y_{i}\Delta x_{\text{j}}\right)\rangle & \langle \left(\Delta y_{i}\Delta y_{j}\right)\rangle & \langle \left(\Delta y_{i}\Delta z_{j}\right)\rangle \\ \langle \left(\Delta z_{i}\Delta x_{j}\right)\rangle & \langle \left(\Delta z_{i}\Delta y_{j}\right)\rangle & \langle \left(\Delta z_{i}\Delta z_{j}\right)\rangle \end{array}\right]$$

Then, the Pearson’s correlation submatrix *R*_*ij*_ is calculated as the covariance submatrix *C*_*ij*_ normalized by the standard deviation

$$R_{ij}=\left[\begin{array}{ccc} \rho_{ij}^{xx} & \rho_{ij}^{xy} & \rho_{ij}^{xz}\\ \rho_{ij}^{yx} & \rho_{ij}^{yy} & \rho_{ij}^{yz}\\ \rho_{ij}^{zx} & \rho_{ij}^{zy} & \rho_{ij}^{zz} \end{array}\right],$$

where

$$\rho_{ij}^{xx}=\langle \left(\Delta x_{i}\Delta x_{j}\right)\rangle /\sqrt{\langle {\left(\Delta x_{i}\right)}^{2}\rangle \langle {\left(\Delta x_{j}\right)}^{2}\rangle },\ldots$$

Here, we define root mean square of *R*_*ij*_ components as *PCC*_*i*,*j*_.

$$PCC_{i,j}=\sqrt{{\left(\rho_{ij}^{xx}\right)}^{2}+{\left(\rho_{ij}^{xy}\right)}^{2}+{\left(\rho_{ij}^{xz}\right)}^{2}+{\left(\rho_{ij}^{yx}\right)}^{2}+{\left(\rho_{ij}^{yy}\right)}^{2}+{\left(\rho_{ij}^{yz}\right)}^{2}+{\left(\rho_{ij}^{zx}\right)}^{2}+{\left(\rho_{ij}^{zy}\right)}^{2}+{\left(\rho_{ij}^{zz}\right)}^{2}}$$ (2)

The RMSF of all heavy atoms was calculated using the g_rmsf module of GROMACS 4.5.5 [53,54]. The calculation of the covariance matrix of all heavy atoms was performed using the g_covar module of GROMACS 4.5.5 and the statistical software R.

These fluctuating indexes were converted to the difference values between the active state R* and the inactive state **R** as formulas 1 and 2, and then, the correlation with G-protein/β-arrestin efficacy was analyzed.

$$\Delta RMSF\left(i\right)=RMSF_{i}^{active}-RMSF_{i}^{inactive}$$ (3)

$$\Delta PCC\left(i,j\right)=PCC_{i,j}^{active}-PCC_{i,j}^{inactive}$$ (4)

Using the analysis of the correlation between ΔRMSF and ligand efficacy, the “fluctuating atoms” were defined as the heavy atoms with a Pearson’s correlation coefficient of more than 0.6. We refer to the fluctuating atoms correlated with the G protein and β-arrestin efficacy as fluctuating atoms^G-protein^ and fluctuating atoms^β-arrestin^, respectively. Using the analysis of the correlation between ΔPCC and ligand efficacy, the “atom–atom couplings” were extracted as the atom pairs with a Pearson’s correlation coefficient of more than 0.6 from all of the heavy atom pairs that included at least one fluctuating atom and that were within a 12 Å distance. We refer to the atom–atom couplings correlated with the G protein and β-arrestin efficacy as atom–atom couplings^G-protein^ and atom–atom couplings^β-arrestin^, respectively.

### Visualization of the G-protein/β-arrestin-linked fluctuating network

VMD was used to visualize the G-protein/β-arrestin-linked fluctuating network [55]. For the atom–atom couplings, we drew a line between atom i and atom j if both atom i and atom j were fluctuating atoms. If atom i was a fluctuating atom and atom j was not a fluctuating atom, we drew a line between atom i and C_α_ atom k of the same residue as atom j to simplify the visualization. The number of pairs is represented by the line width. We only show the lines between atom i and C_α_ atom k if the two atoms are located in different secondary structures (helix or loop), which are likely to be characteristic of the fluctuating network. It is important to note that these were applied only for visualization purposes, while for the analysis, all heavy atoms were considered.

### Calculation for predicting ligand efficacy and bias

To predict the ligand efficacy of G protein activity, we calculated the average ΔRMSF of all C_α_ atoms in the lower half of helix 6 (Cys265^6.27^–Trp286^6.48^), in which the fluctuating atoms^G-protein^ were particularly concentrated, and the average ΔPCC of all C_α_ atom pairs between helix 6 (Cys265^6.27^–Trp286^6.48^) and helix 3 (Val117^3.36^–Arg131^3.50^), in which the highest number of atom–atom couplings^G-protein^ was detected.

For β-arrestin activity, we calculated the average ΔRMSF of C_α_ atoms using the residues in helix 1 (Gly50^1.49^, Val52^1.51^, Leu53^1.52^, and Thr56^1.55^) and helix 7 (Pro323^7.50^–Ile325^7.52^), which include the fluctuating atoms^β-arrestin^ and are in the center of the β-arrestin-linked fluctuating network. We used the average ΔPCC of all C_α_ atom pairs between helix 1 (Gly50^1.49^, Val52^1.51^, Leu53^1.52^, and Thr56^1.55^) and helix 7 (Ser319^7.46^–Ile325^7.52^), in which the highest number of atom–atom couplings^β-arrestin^ was detected.

## Results

### Fluctuation of apo receptors within a microsecond

**Fig 2:**
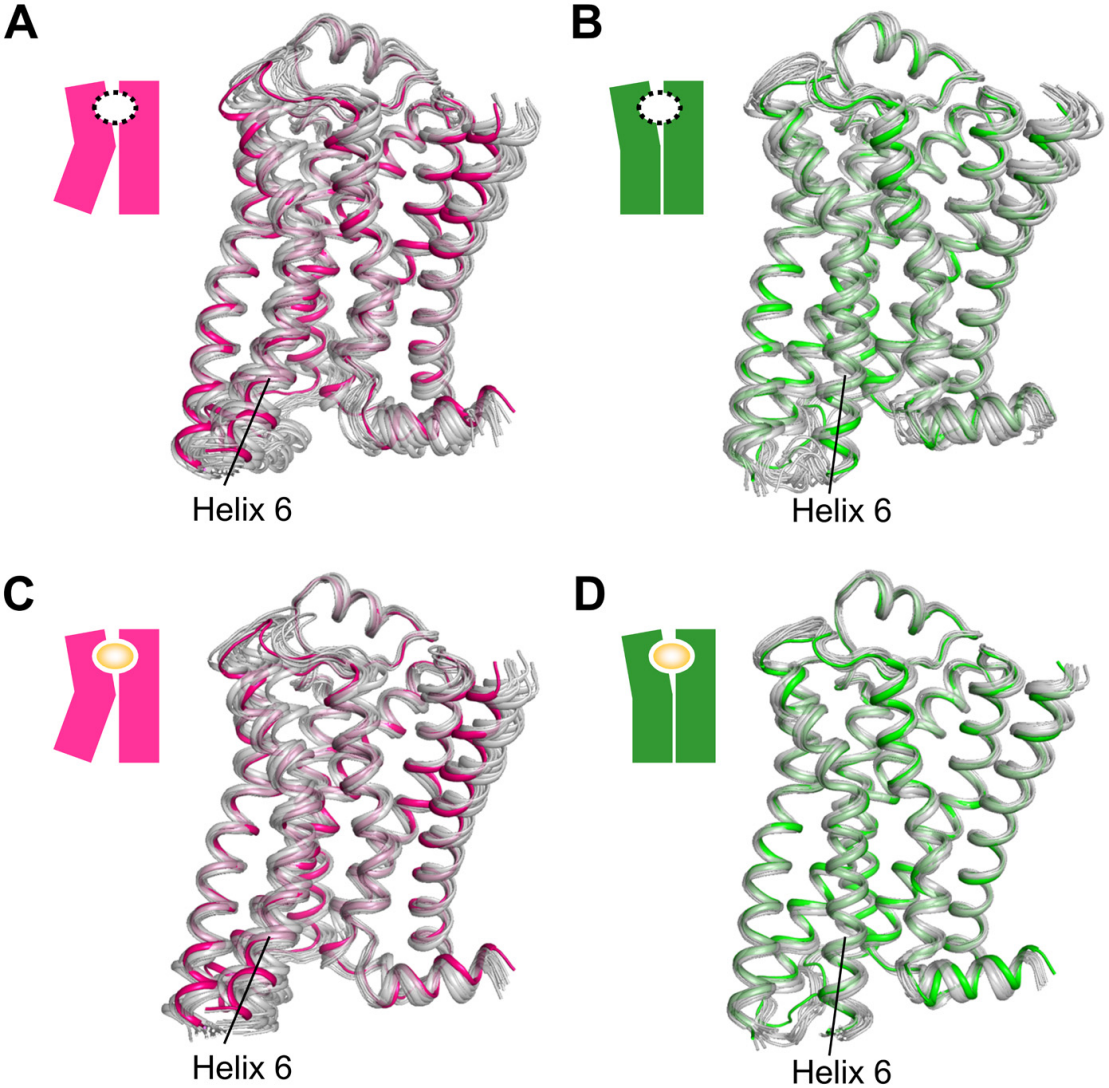
β_2_AR structures during our MD simulation. The structures of β_2_AR during our MD simulation (gray) are superimposed on the crystal structures of the inactive state **R** (PDB ID: 2RH1; green) or the G-protein-active state R* (PDB ID: 3SN6; magenta). (A) The snapshots from 1-μs MD simulation of the apo-receptor starting from the R* state at 100 ns intervals. (B) The snapshots from 1-μs MD simulation of the apo-receptor starting from the **R** state at 100 ns intervals. (C) The 14 averaged structures of ligand-bound receptors starting from the R* state. D. The 14 averaged structures of ligand-bound receptors starting from the **R** state.

We performed a 1-μs MD simulation of apo **R** and R* to confirm the time scale of the conformational change. During the 1-μs simulations, the overall structures of both the inactive state **R** and G-protein-active state R* did not undergo large conformational changes, as indicated by the backbone RMSD to the initial structures (mean ± SD) being maintained within 1.3 ± 0.1 Å for **R** and 2.0 ± 0.2 Å for R* (Fig 2A and 2B and S3 Fig). Furthermore, the distance between helix 3 (Arg131^3.50^ C_α_) and helix 6 (Leu272^6.34^ C_α_), which is the region that differs the most between the active and inactive crystal structures, was retained at 7.7 ± 0.4 Å for **R** and 14.8 ± 0.6 Å for R*. The slightly larger standard deviation for R* than for **R** indicates that the fluctuation of R* is larger than that of **R** in helix 3 and/or helix 6. Other well-studied regions [25,30] also showed negligible conformational differences between the beginning and the end of the simulation (S3 Fig). Thus, we were not able to detect a large portion of the transition pathway between R* and **R** during 1 μs.

### Fluctuation of ligand-bound receptors within sub-microseconds

In order to analyze the short-term fluctuation, we performed sub-microsecond MD simulations of **R** and R* complexed individually with 14 diverse β_2_AR ligands (Table 1 and S1 Fig). As we expected, for all ligands, the ligand-bound **R** retained almost the same conformations as the initial structure, that is, 1.1–1.4 Å RMSDs for the backbone atoms (Fig 2D and S4 Fig). In the ligand-binding site, each ligand underwent a slight “induced fit,” with 0.9–1.3 Å RMSDs for C_α_ atoms within 4 Å from the ligand. In the G-protein-binding site, the distance between helix 3 (Arg131^3.50^ C_α_) and helix 6 (Leu272^6.34^ C_α_) remained unchanged in all simulations; the mean ± SD for the average distance of each complex was 7.8 ± 0.1 Å (S4 Fig). Likewise, for all ligands, the ligand-bound R* did not undergo a significant conformational change in the backbone, with RMSDs of 1.6–2.2 Å, RMSDs for the ligand-binding site of 1.0–1.5 Å, and the helix6–helix3 average distance of 14.4 ± 0.6 Å (mean ± SD) (Fig 2C and S4 Fig). For any of the tested ligands, notable conformational changes of **R** or R* were not indicated by average distances and RMSDs in the sub-microsecond simulations.

### G-protein-linked fluctuating network

**Fig 3:**
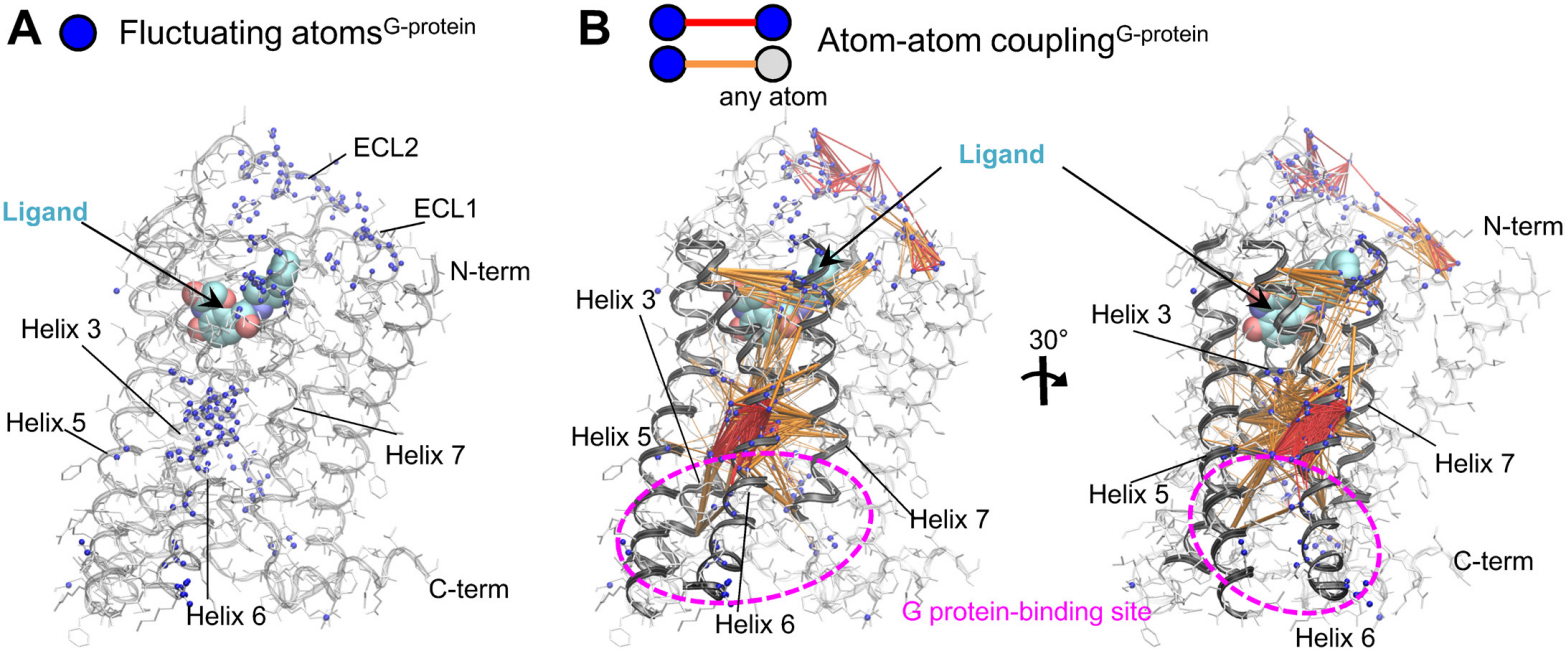
G-protein-linked fluctuating network of β_2_AR. (A) Fluctuating atoms^G-protein^, which have a Pearson’s correlation coefficient of more than 0.6 between their ΔRMSF and G protein efficacy, are indicated by blue spheres. (B) Atom–atom couplings^G-protein^ were extracted from any pairs containing at least one fluctuating atom^G-protein^ by correlation analysis between ΔPCC and G protein efficacy. The atom–atom pairs with more than a 0.6 Pearson’s correlation coefficient and within 12 Å are connected by lines. In the cases where both atoms are fluctuating atoms^G-protein^, the lines are colored red. In cases where one of the two atoms is a fluctuating atom^G-protein^, the lines are colored orange (see the Methods section). The G-protein-binding site is shown with a dashed magenta ellipse.

We identified the G-protein-linked fluctuating network using the short-term fluctuation difference between R* and **R** for each ligand, which was calculated from the trajectories of sub-microsecond MD simulations. The G-protein-linked fluctuating network consists of the “fluctuating atoms^G-protein^” and the “atom–atom couplings^G-protein^” (Fig 3 and S7 Fig).

Almost 200 atoms were determined to be fluctuating atoms^G-protein^, which have a correlation between their ΔRMSF (Eq 3) and G protein efficacy (S1 File). Most of these atoms are located at helices 3, 5, 6, and 7 and ECL1 and ECL2. The fluctuating atoms^G-protein^ were particularly concentrated on the lower half of helix 6, which is the region that differs the most between the active and inactive crystal structures, including Ala271^6.33^, Leu275^6.37^, and Ile278^6.40^–Cys285^6.47^. The atom–atom couplings^G-protein^ were extracted from any pairs containing at least one fluctuating atom^G-protein^ by correlation analysis between ΔPCC (Eq 4) and G protein efficacy (S2 File). In contrast to the isolated atom–atom couplings^G-protein^ in ECL1/2, the atom–atom couplings^G-protein^ in helices 3, 5, 6, and 7 were found across multiple helices as well as within each helix (Fig 3B). In particular, a large number of atom–atom couplings^G-protein^ were detected across helix 3–helix 6, for which interhelical angles change significantly between the inactive and active structures [13,56]. Interestingly, we found that the G-protein-linked fluctuating network ranged from the ligand-binding site to the upper part of the G-protein binding site through the connector region of helices 3, 5, 6, and 7. In addition, this dense network seems to involve the lower part of the G-protein binding site and the ECL1/2. In other words, our results show that the degree of the amplitude and the connection in this continuous network are responsible for G protein efficacy.

### β-Arrestin-linked fluctuating network

**Fig 4:**
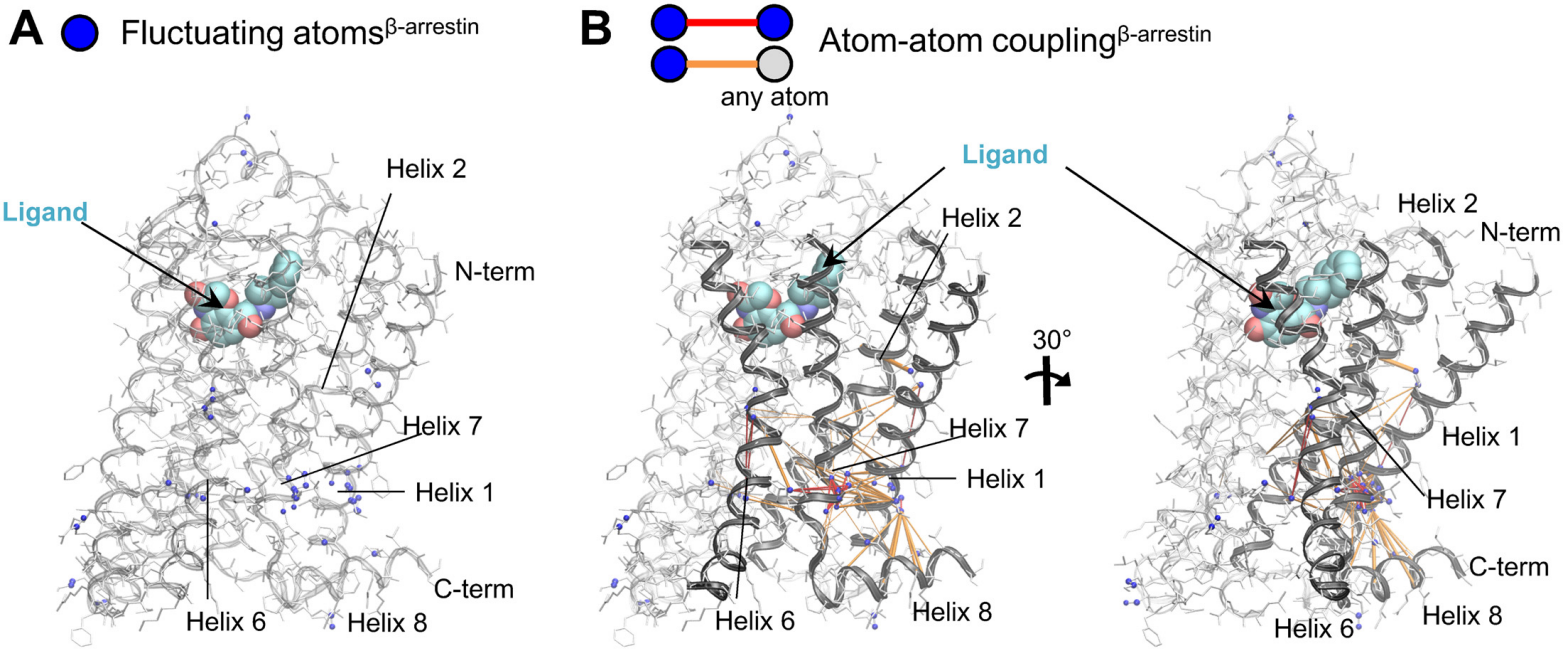
β-Arrestin-linked fluctuating network of β_2_AR. (A) Fluctuating atoms^β-arrestin^, which have a Pearson’s correlation coefficient of more than 0.6 between their ΔRMSF and β-arrestin efficacy, are indicated by blue spheres. (B) Atom–atom couplings^β-arrestin^ were extracted from any pairs containing at least one fluctuating atom^β-arrestin^ by correlation analysis between ΔPCC and β-arrestin efficacy. Lines are depicted in the same manner as in Fig 3.

The β-arrestin-active R** state, which leads to the blockade of G-protein signaling and initiation of β-arrestin signaling, is generally thought to be the state in which GPCRs bind to β-arrestin after the transition from the inactive **R** state to the G-protein-active R* state [57]. If R** transitions from R*, an analysis using R* and R** would be ideal to determine the β-arrestin-linked fluctuating network. However, the crystal structure of R** has not been determined to date. Instead, because the fluctuation of R* is expected to intrinsically include a part of the fluctuation related to the transition into R**, we extracted the β-arrestin-linked fluctuating network using **R** and R*. In this study, all of the ligands that show significant β-arrestin efficacy have potent G protein efficacy (Table 1). It means that the component of β-arrestin-linked fluctuating network could be found out from the mixture of various fluctuation by analyzing the correlation between ΔRMSF/ΔPCC using **R** and R* and β-arrestin efficacy, instead of G protein efficacy (Fig 4 and S8 Fig).

A much smaller number of atoms (about 50) were determined to be fluctuating atoms^β-arrestin^, indicating a correlation between their ΔRMSF and β-arrestin efficacy (Pearson’s correlation coefficient more than 0.6), than were determined to be fluctuating atoms^G-protein^ (S3 File). These atoms localized at helix 1 (Val44^1.43^, Gly50^1.49^, Val52^1.51^, Val53^1.52^, Thr56^1.55^) and helix 7 (Pro323^7.50^, Leu324^7.51^, Ile325^7.52^). The atom–atom couplings^β-arrestin^, which correlate with β-arrestin efficacy (Pearson’s correlation coefficient more than 0.6), connected adjacent helices, including helix 1–helix 7/8 and helix 7–helix 2/6/8 (S4 File).

Taken together, the β-arrestin-linked fluctuating network is located in the specific narrow region that consists of helices 1 and 7 and the adjacent helices 2, 6, and 8. Interestingly, the center of this network is formed by the NPxxY motif in helix 7, which is known to be important in the GPCR activation and desensitization processes [5,58].

### Prediction of ligand efficacy and bias

**Fig 5:**
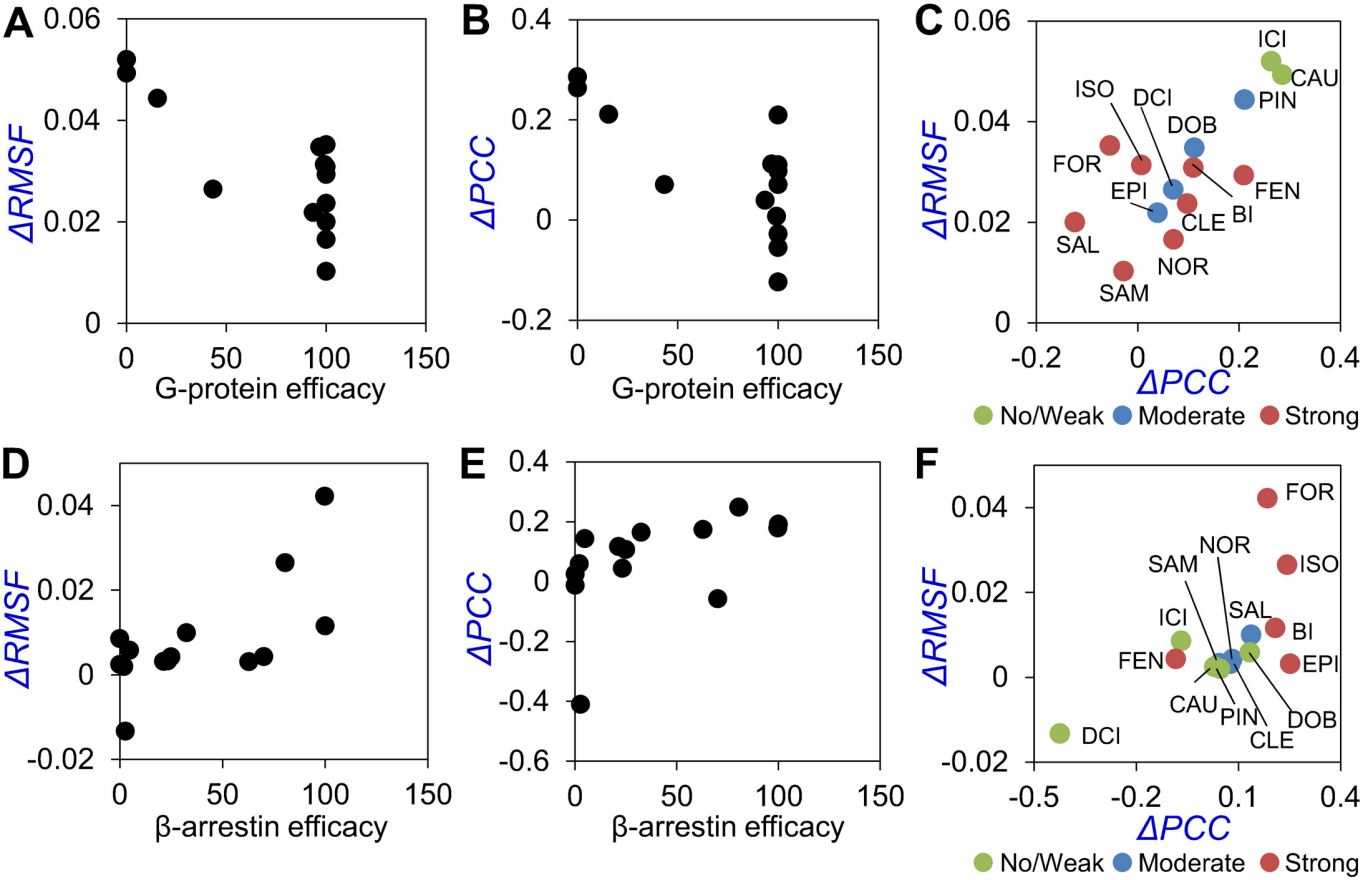
Plots of the mean ΔRMSF and ΔPCC against G protein efficacy. The mean ΔRMSF and ΔPCC using C_α_ atoms, ΔRMSF(C_α_) and ΔPCC(C_α_), were plotted against G protein efficacy (A, B, C) and β-arrestin efficacy (D, E, F). The 14 ligands are shown as circles. (A) Plot of the mean ΔRMSF(C_α_) of helix 6, on which the fluctuating atoms^G-protein^ were particularly concentrated, against the G protein efficacy from a previous study [27]. (B) Plot of the mean ΔPCC(C_α_) between helix 3–helix 6, in which a large number of atom–atom couplings^G-protein^ were detected, against G protein efficacy. (C) Plot of the mean ΔRMSF(C_α_) of helix 6 against the mean ΔPCC(C_α_) between helix 3–helix 6. Red: Ligands with a strong G protein efficacy that have larger E_max_ values than isoprenaline; Blue: Ligands with a moderate G protein efficacy that have smaller E_max_ values than isoprenaline; Green: Ligands with no/weak efficacy. (D) Plot of the mean ΔRMSF(C_α_) of specific residues in helix 1 and helix 7 against the β-arrestin efficacy from a previous study [27]. (E) Plot of the mean ΔPCC(C_α_) of specific residue pairs between helix 1 and helix 7 against β-arrestin efficacy. (F) Plot of the mean ΔRMSF(C_α_) against the mean ΔPCC(C_α_). Red: Ligands with a strong β-arrestin efficacy (E_max_ ≥50); Blue: Ligands with a moderate β-arrestin efficacy (E_max_ 10–50); Green: Ligands with no/weak β-arrestin efficacy (E_max_ <10).

The G-protein-/β-arrestin-linked fluctuating network was identified by analyzing ΔRMSF and ΔPCC using sub-microsecond MD simulations. These relations between the ligand efficacy of G protein/β-arrestin and GPCR fluctuation can potentially overcome the challenge of designing GPCR drugs with appropriate efficacy and bias. We evaluated whether the fluctuations quantified by both ΔRMSF and ΔPCC enable the prediction of the efficacy and bias for drug candidates (Fig 5). Here, we calculated the mean of ΔRMSF and ΔPCC using C_α_ atoms (see the Methods section), instead of all heavy atoms, in order to obtain an outlook for the future application to not only β_2_AR but also other aminergic GPCRs.

For the prediction of G protein efficacy, the mean score was calculated for the concentrated region of the G-protein-liked fluctuating network, which is all C_α_ atoms in the lower half of helix 6 for ΔRMSF and all pairs of C_α_ atoms in the lower half of helix 6–helix 3 for ΔPCC. The quantitative values of the short-term fluctuation difference for 14 known ligands are shown with the G protein efficacy data in Fig 5A–5C. The plots indicate that ΔRMSF and ΔPCC are moderately correlated with G protein efficacy. We also confirmed a moderate correlation in the plot of both ΔRMSF and ΔPCC for C_α_ atoms (Fig 5C), which shows the potential for the future application using other heavy atoms and combination of ΔRMSF and ΔPCC. Two ligands with negligible G protein efficacies (carazolol and ICI-119551) had large ΔRMSF and ΔPCC values, and eight ligands with strong G protein efficacies, which had larger E_max_ values than isoprenaline in a previous study [27] (Table 1), tended to have small ΔRMSF and ΔPCC values. Four ligands with moderate G protein efficacies, which had smaller E_max_ values than isoprenaline (Table 1), had intermediate ΔRMSF and ΔPCC values.

The β-arrestin-linked fluctuating network extracted from **R** and R* was identified as a specific narrow region mainly consisting of helices 1 and 7. As with G protein efficacy, the quantitative values of short-term fluctuation differences are shown with the β-arrestin efficacy data in Fig 5D–5F. A plot of the mean ΔRMSF for β-arrestin efficacy (Fig 5D) shows a stronger correlation than that of ΔPCC or both ΔRMSF and ΔPCC. The ligands with stronger β-arrestin efficacies tend to have larger ΔRMSF values.

## Discussion

Our practical method to predict the efficacy and bias of GPCR signaling will be useful for designing drugs that are more precise. Furthermore, the G-protein- and β-arrestin-linked fluctuating networks, which are bases of this method, will be helpful to understand how large-scale conformational changes are triggered by ligand—protein interactions.

The G-protein-linked fluctuating network covers most of the regions shown to undergo conformational change in previous long timescale MD simulations. The network contains the ligand-binding site and the G-protein-binding site. We compared the G-protein-linked fluctuating network with the results of a previous study of the transition pathway from R* to **R** using a total of more than 650 μs MD simulation [25] (Fig 3 and S7 Fig).

For the ligand-binding site, the G-protein-linked fluctuating network includes residues on helix 5, and they couple with the connector region (S1 Text). This is consistent with previous studies indicating that helix 5 movement was the primary change upon binding. In addition, the G-protein-linked fluctuating network involves not only helix 5 residues but also more than half of the ligand-contact residues in helices 3, 6, and 7 [13], for example, Phe289^6.51^, which interacts with the aromatic ring of ligand by a π–π interaction, Val114^3.33^, Val117^3.36^, Tyr308^7.35^, and Ile309^7.36^, which have van der Waals contact with a ligand, and Asn312^7.39^, which forms hydrogen bonds with a ligand. This result shows that the fluctuating network is controlled not only by interaction between a few residues in helix 5 and the ligand but also by comprehensive interaction with multiple residues in helices 3, 5, 6, and 7. Between the ligand-binding and G-protein-binding sites, there is a tightly coupled “core” of the G-protein-linked fluctuating network formed by helices 3, 5, and 6. Ile121^3.40^ and Phe282^6.44^, which are located at the center of the core, show relatively large conformational differences between R* and **R**, and they were determined to be the main residues in the connector region in a previous study [25]. Here, we found that the fluctuation of Ile121^3.40^ and Phe282^6.44^ is not isolated but tightly couples with adjacent fluctuating atoms^G-protein^. The core of the fluctuating atoms^G-protein^ couples with a broad region of helices 3, 5, 6, and 7, including the bottom of the ligand-binding site and the top of the G-protein-binding site, which demonstrates how the conformational changes of Ile121^3.40^ and Phe326^6.44^ are induced from the ligand-binding site and transfer the signal to the G-protein-binding site (S1 Text). In the G-protein-binding region, Tyr219^5.58^ and Tyr326^7.53^ are characteristic residues that define the receptor state [25]. Tyr326^7.53^ is a member of the G-protein-linked fluctuating network. Tyr219^5.58^ itself is not in the network, but all of its contacting residues are included in the network (S1 Text). Furthermore, the G-protein-linked fluctuating network involves the G-protein-contact residues [13], such as Arg131^3.50^, Ala271^6.33^, and Leu275^6.37^. The highly conserved amino acid residues on helices 3, 5, 6, and 7 in the G-protein-linked fluctuating network, which shows more than 60% amino acid sequence similarity among aminergic GPCRs, suggest that the trigger mechanism could be a common feature in aminergic GPCRs.

In addition, the β-arrestin-linked fluctuating network revealed here could provide structural insights into the conformational change that induces β-arrestin signaling. The core of the β-arrestin-linked fluctuating network is the NPxxY motif in helix 7 and the adjacent helix 1 (Fig 4 and S8 Fig). This is consistent with previous studies that reported the perturbation of transmembrane helix 7 by β-arrestin effective ligands [23,24] and that revealed the primary phosphorylation of a region directly connected to helix 7 on helix 8 by GPCR kinases [59], which is a prerequisite for β-arrestin binding. Moreover, our results reveal the close relation between β-arrestin efficacy and local fluctuation, including coupling between the NPxxY motif and surrounding residues in helices 1, 2, 6, and 8, and that these specific fluctuations might trigger the initial conformational change for β-arrestin signaling. However, the β-arrestin-linked fluctuating network using **R** and R* states does not contain any atom in the ligand-binding site, as opposed to the G-protein-linked fluctuating network. Although the fluctuation of the G-protein-active R* state is likely to include the fluctuation related to the transition into the β-arrestin-active R** state, it might not be sufficient for extracting the complete network because of the noise caused by other fluctuations, including thermal fluctuation, and the largest part of the fluctuating network was likely detected in this study. The β-arrestin-linked fluctuating network analysis using the β-arrestin-active R** state would clarify the complete network ranging from the ligand-binding site to the β-arrestin-binding site.

The β_2_AR is known to show not only the typical states such as **R**, R*, and R**, but also various intermediate states. The association between these intermediate states and the G-protein/β-arrestin-linked fluctuating networks could be revealed by much larger ensembles of the simulations and the simulations starting from the intermediate states. In the MD simulations of apo **R** and R*, the fluctuation patterns of the distances and the RMSDs monitored in 1-μs simulation are almost same to the first 150 ns simulations (Fig 2 and S3 Fig), which suggests that 150 ns simulation includes nearly as much information about fluctuation as 1-μs simulation. In addition, because the G-protein-linked fluctuating network is consistent with previous simulations of the transition pathway from R* to **R** through an intermediate state [25], common fluctuations might be observed in this intermediate state. The further association between the intermediate states and the fluctuations will be clarified in our future work using the various intermediate states.

We propose a method using the G-protein/β-arrestin-linked fluctuating network to predict ligand efficacy and bias. In order to confirm the potential as a prediction method, we analyzed receptors bound with a neutral antagonist, alprenolol, and no ligand, apo, in the same manner as receptors with 14 different ligands. The means of ΔRMSF/ΔPCC to predict G protein efficacy were 0.046/0.31 for alprenolol and 0.045/0.16 for no ligand, which were close to those of the inverse agonists and the weak partial agonist pindolol. The means of ΔRMSF to predict β-arrestin efficacy were 0.017 for alprenolol and 0.0071 for no ligand, which were acceptable values indicating no/weak β-arrestin efficacy. These validations showed that the simple mean ΔRMSF and/or ΔPCC values of the C_α_ atom in the efficacy-linked fluctuating network could roughly explain the experimental values. More specific indicators defined by other heavy atoms and/or specific residues and further validations could be used to predict ligand efficacy and bias.

It is should be noted that this study has been focused on analyzing the overall relationships between the fluctuation and the efficacies by using all runs of 14 ligands. To our knowledge, this is the first study that the fluctuations with so many ligands have been analyzed all together. These findings would facilitate future efforts to characterize an individual ligand-specific fluctuation by further simulations of the individual ligand-bound receptor under many different conditions [25].

In conclusion, we performed all-atom MD simulations of active (R*) and inactive (**R**) β_2_AR complexed with well-characterized ligands that have various G-protein/β-arrestin efficacies and measured the conformational fluctuation at the sub-microsecond scale. We focused on analyzing the fluctuation difference between R* and **R**, instead of the transition pathway between them, and extracted the G-protein/β-arrestin-linked fluctuating network without prior knowledge of the transition pathway. The G-protein-linked fluctuating network extends from the ligand-binding site to the G-protein binding site through the transmembrane helices 3, 5, 6, and 7 centered by the connector region. On the other hand, the β-arrestin-linked fluctuating network is located in the specific narrow region that consists of the NPxxY motif in helix 7 and the adjacent helix, which is consistent with previous studies that reported the perturbation of helix 7 by β-arrestin effective ligands. Finally, we showed that fluctuations quantified by both ΔRMSF and ΔPCC could be used to predict the efficacy and bias of candidate compounds during drug discovery research. It is worth noting that the time cost of our approach that characterizes the initial fluctuation is two orders of magnitude smaller than an approach that simulates the transition pathway. Our approach that focuses on the initial fluctuation might be a powerful and broadly applicable method for studying many biological systems.

## Supporting Information

S1 Fig Structure and efficacy of 14 β_2_AR ligands. (A) The structure of 14 β_2_AR ligands. (B) Plot of the G protein and β-arrestin efficacy for 14 ligands.
(PDF)

S2 Fig The binding pose of each 14 ligands. The snapshots of the inactive simulation after the 20 ns NPT equilibration. Ligands are shown by green stick model, and the residues within 4 Å from the ligand are shown by line. The binding poses of the active simulation are almost same as those of the inactive simulation.
(PDF)

S3 Fig Time series of structural changes in simulation of apo receptors. The results of G-protein-active R* state and inactive **R** state are colored red and green, respectively. (A) Backbone RMSD of all region relative to the initial crystal structure (PDB ID: 2RH1, 3SN6). (B) Helix 3–helix 6 distance, which is monitored by Arg131^3.50^–Leu272^6.34^ C_α_ atom distance. (C) RMSD from the inactive crystal structure (PDB ID: 2RH1) of the nonsymmetrical, heavy atoms in Ile121^3.40^ and Phe282^6.44^. (D) RMSD of NPxxY region (residues Asn322^7.45^–Cys327^7.54^) backbone atoms relative to the inactive crystal structure.
(PDF)

S4 Fig Time series of structural changes in simulation of ligand-bound receptors. The results of G-protein-active R* state and inactive **R** state are colored red and green, respectively. (A) Backbone RMSD of all region relative to the initial crystal structure (PDB ID: 2RH1, 3SN6). (B) Helix 3–helix 6 distance, which is monitored by Arg131^3.50^–Leu272^6.34^ C_α_ atom distance.
(PDF)

S5 Fig Plots of RMSF and ΔRMSF against all of the heavy atoms. The RMSF of G-protein-active R* state and inactive **R** state and ΔRMSF are colored red, green and blue, respectively. The region of helices are shown as orange bar.
(PDF)

S6 Fig Plots of ΔPCC for all of the heavy atom—heavy atom pairs. The positive value and negative values are colored blue and red, respectively. The region of helices are shown as orange bar.
(PDF)

S7 Fig Detail views of the G-protein-linked fluctuating network. The residues that constitute the G-protein-linked fluctuating network are shown by cyan stick model. The fluctuating atoms^G-protein^ are shown by blue spheres. The atom–atom couplings^G-protein^ are shown by red or orange dashed line with the same coloring as Fig 3B. (A) Overall view of the G-protein-linked fluctuating network in the same position as right view of Fig 3B. (B) Side view of the ligand-binding site. (C) Bottom view from the intracellular region of the G-protein-binding site. (D) Side view of the connector region.
(PDF)

S8 Fig Detail views of the β-arrestin-linked fluctuating network. The residues that constitute the β-arrestin-linked fluctuating network are shown by cyan stick model. The fluctuating atoms^β-arrestin^ are shown by blue spheres. The atom–atom couplings^β-arrestin^ are shown by red or orange dashed line with the same coloring as Fig 4B. (A) Overall view of the β-arrestin-linked fluctuating network in the same position as left view of Fig 4B. The β-arrestin-binding site are shown by dashed magenta ellipse. (B) Side view of the β-arrestin-binding site. (C) Bottom view from the intracellular region of the β-arrestin-binding site in the same position as S7C Fig.
(PDF)

S1 File The fluctuating atoms^G-protein^. All of the residues that include the fluctuating atoms^G-protein^ are listed. The number of the fluctuating atoms^G-protein^ is shown in the right column.
(XLS)

S2 File The atom–atom couplings^G-protein^. (A) All of the residue—residue pairs that include the atom–atom couplings^G-protein^ between two fluctuating atoms^G-protein^ are listed. The number of the atom–atom couplings^G-protein^ in the residue—residue pair is shown in the right column. (B) All of the residue—residue pairs that include the atom–atom couplings^G-protein^ between a fluctuating atom^G-protein^ and any other atom in different helix/loop are listed.
(XLS)

S3 File The fluctuating atoms^β-arrestin^. All of the residues that include the fluctuating atoms^β-arrestin^ are listed. The number of the fluctuating atoms^β-arrestin^ in the residue is shown in the right column.
(XLS)

S4 File The atom–atom couplings^β-arrestin^. (A) All of the residue—residue pairs that include the atom–atom couplings^β-arrestin^ between the two fluctuating atoms^β-arrestin^ are listed. The number of the atom–atom couplings^β-arrestin^ in the residue—residue pair is shown in the right column. (B) All of the residue—residue pairs that include the atom–atom couplings^β-arrestin^ between a fluctuating atoms^β-arrestin^ and any other atom in different helix/loop are listed.
(XLS)

S1 Text Supplementary discussion. (DOC)
