# Supplementary material for: G-Protein/β-Arrestin-Linked Fluctuating Network of G-Protein-Coupled Receptors for Predicting Drug Efficacy and Bias Using Short-Term Molecular Dynamics Simulation
Source: PLoS One. 2016 May 17;11(5):e0155816. doi: 10.1371/journal.pone.0155816 (PMC4871340; doi:10.1371/journal.pone.0155816)

A

Abbre-viated name	Name	Structure	Enan-tiomer
BI	BI-167107	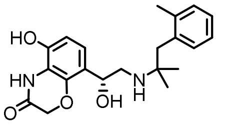	R
ISO	Isoprenaline	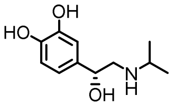	R
FEN	Fenoterol	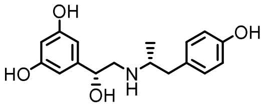	R, R
FOR	Formoterol	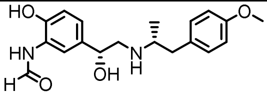	R, R
SAM	Salmeterol	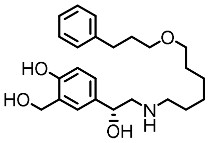	R
CLE	Clenbuterol	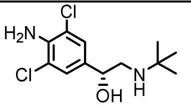	R
SAL	Salbutamol	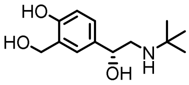	R
NOR	Norepinephrine	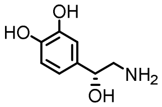	R
DOB	Dobutamine	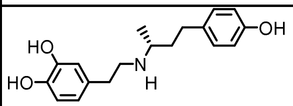	R
EPI	Epinephrine	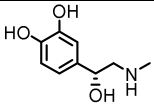	R
DCI	Dichloro-isoproterenol	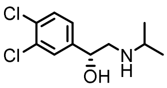	R
PIN	Pindolol	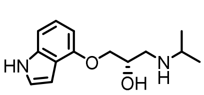	S
CAU	Carazolol	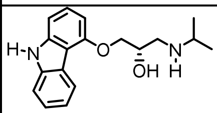	S
ICI	ICI-118551	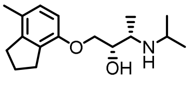	S, S

B

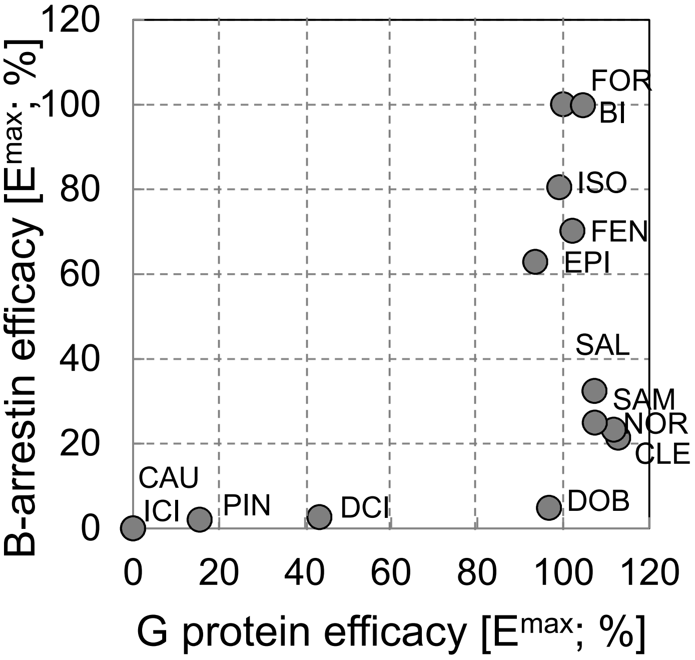

Supplement: S1 Fig — (A) The structure of 14 β2AR ligands. (B) Plot of the G protein and β-arrestin efficacy for 14 ligands. (PDF) [file pone.0155816.s001.pdf]
