# Supplementary figures and images for: G-Protein/β-Arrestin-Linked Fluctuating Network of G-Protein-Coupled Receptors for Predicting Drug Efficacy and Bias Using Short-Term Molecular Dynamics Simulation
Source: PLoS One. 2016 May 17;11(5):e0155816. doi: 10.1371/journal.pone.0155816 (PMC4871340; doi:10.1371/journal.pone.0155816)

BI

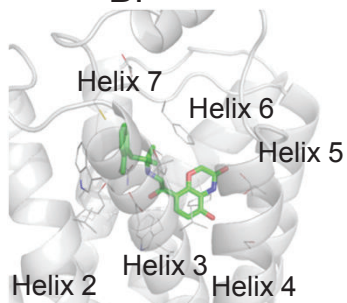

ISO

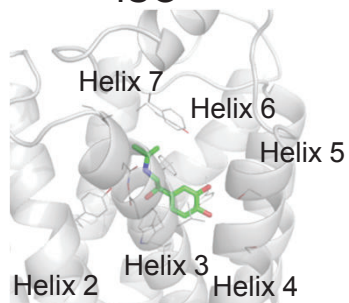

FEN

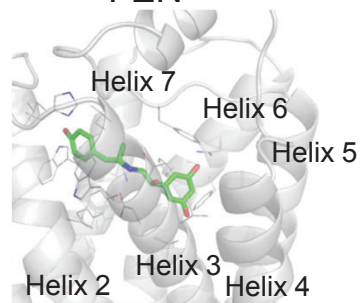

FOR

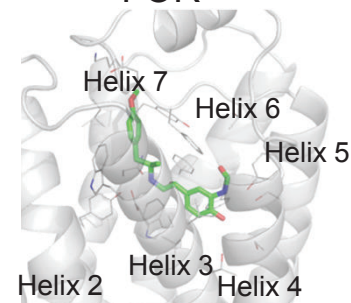

SAM

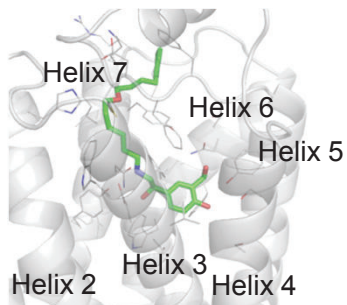

CLE

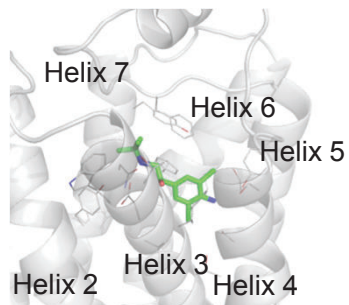

SAL

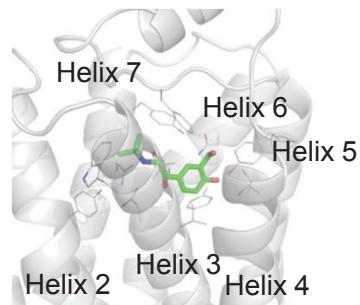

NOR

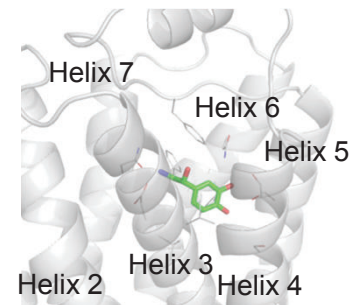

DOB

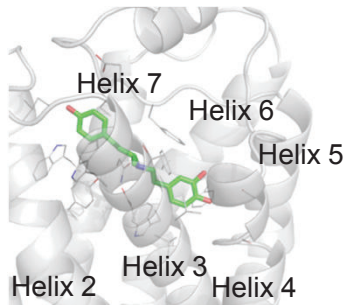

EPI

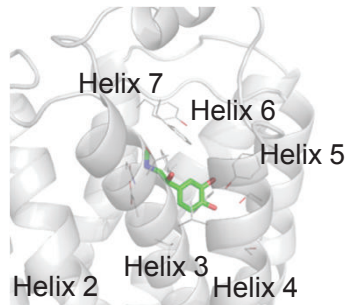

DCI

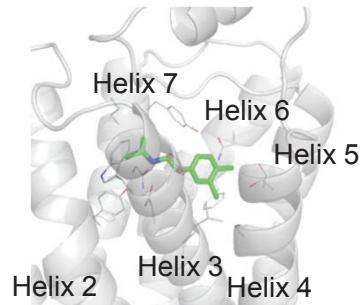

PIN

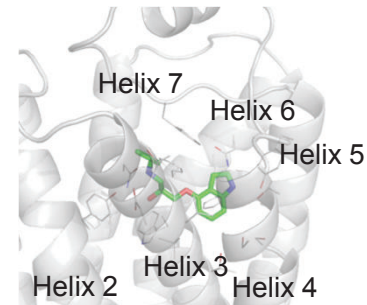

CAU

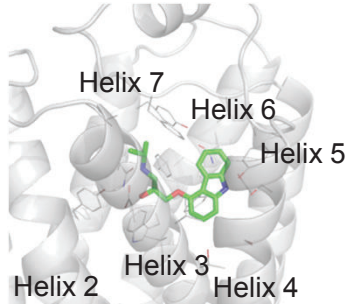

ICI

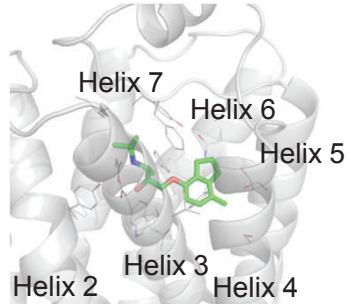

Supplement: S2 Fig — The snapshots of the inactive simulation after the 20 ns NPT equilibration. Ligands are shown by green stick model, and the residues within 4 Å from the ligand are shown by line. The binding poses of the active simulation are almost same as those of the inactive simulation. (PDF) [file pone.0155816.s002.pdf]

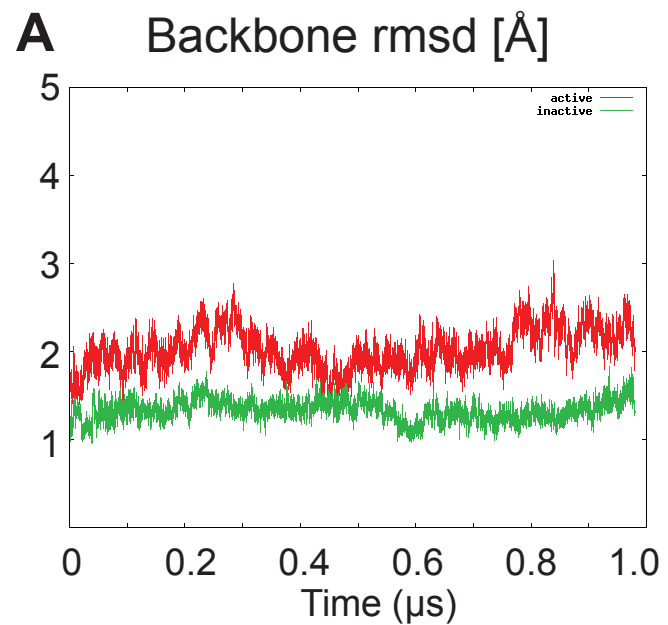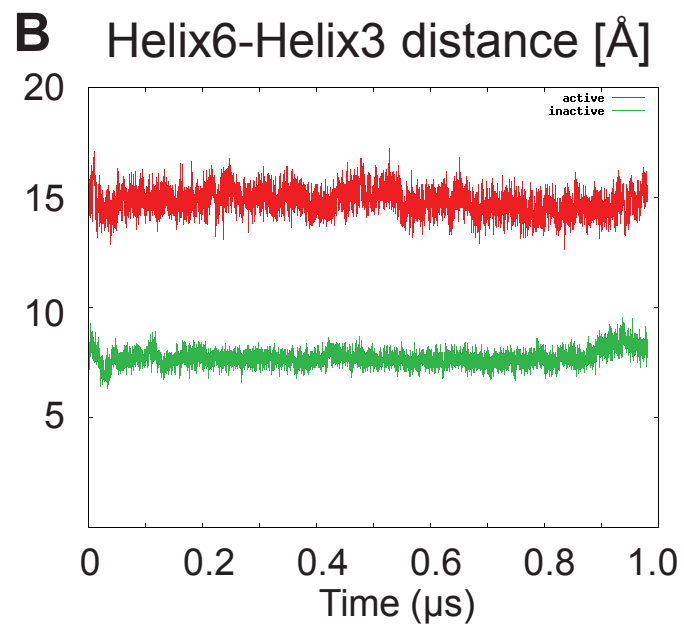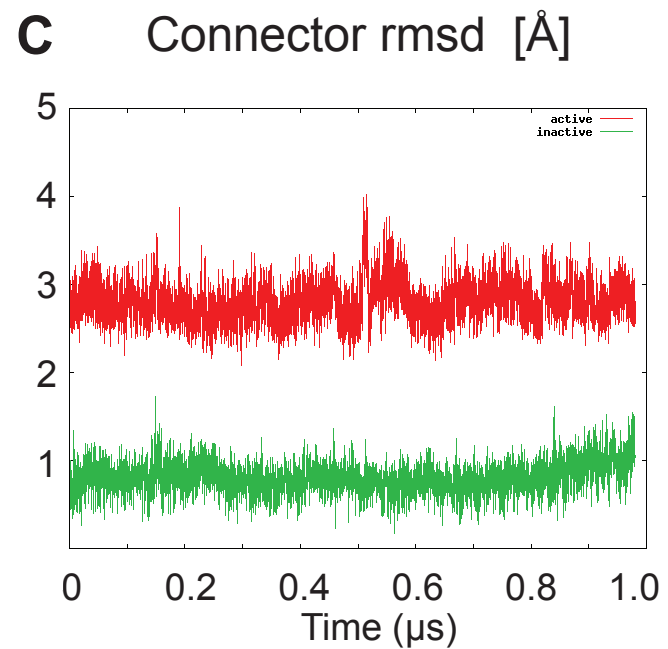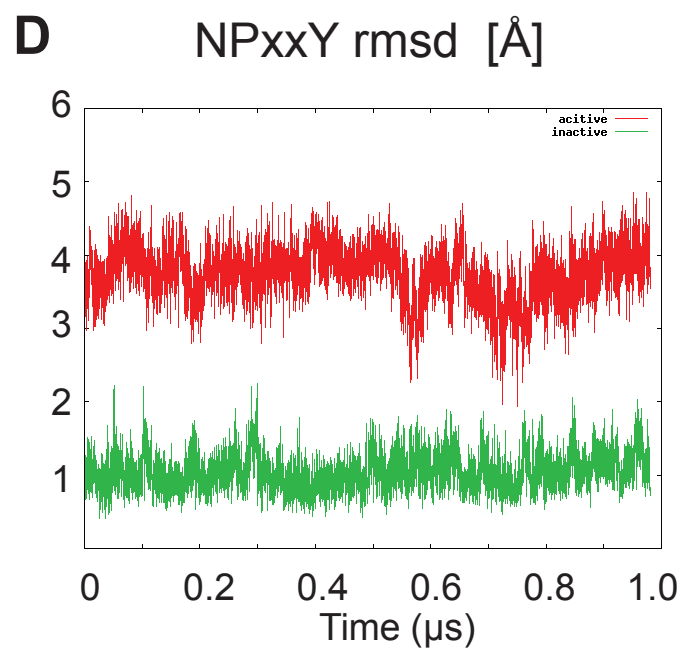

Supplement: S3 Fig — The results of G-protein-active R* state and inactive R state are colored red and green, respectively. (A) Backbone RMSD of all region relative to the initial crystal structure (PDB ID: 2RH1, 3SN6). (B) Helix 3–helix 6 distance, which is monitored by Arg1313.50–Leu2726.34 Cα atom distance. (C) RMSD from the inactive crystal structure (PDB ID: 2RH1) of the nonsymmetrical, heavy atoms in Ile1213.40 and Phe2826.44. (D) RMSD of NPxxY region (residues Asn3227.45–Cys3277.54) backbone atoms relative to the inactive crystal structure. (PDF) [file pone.0155816.s003.pdf]

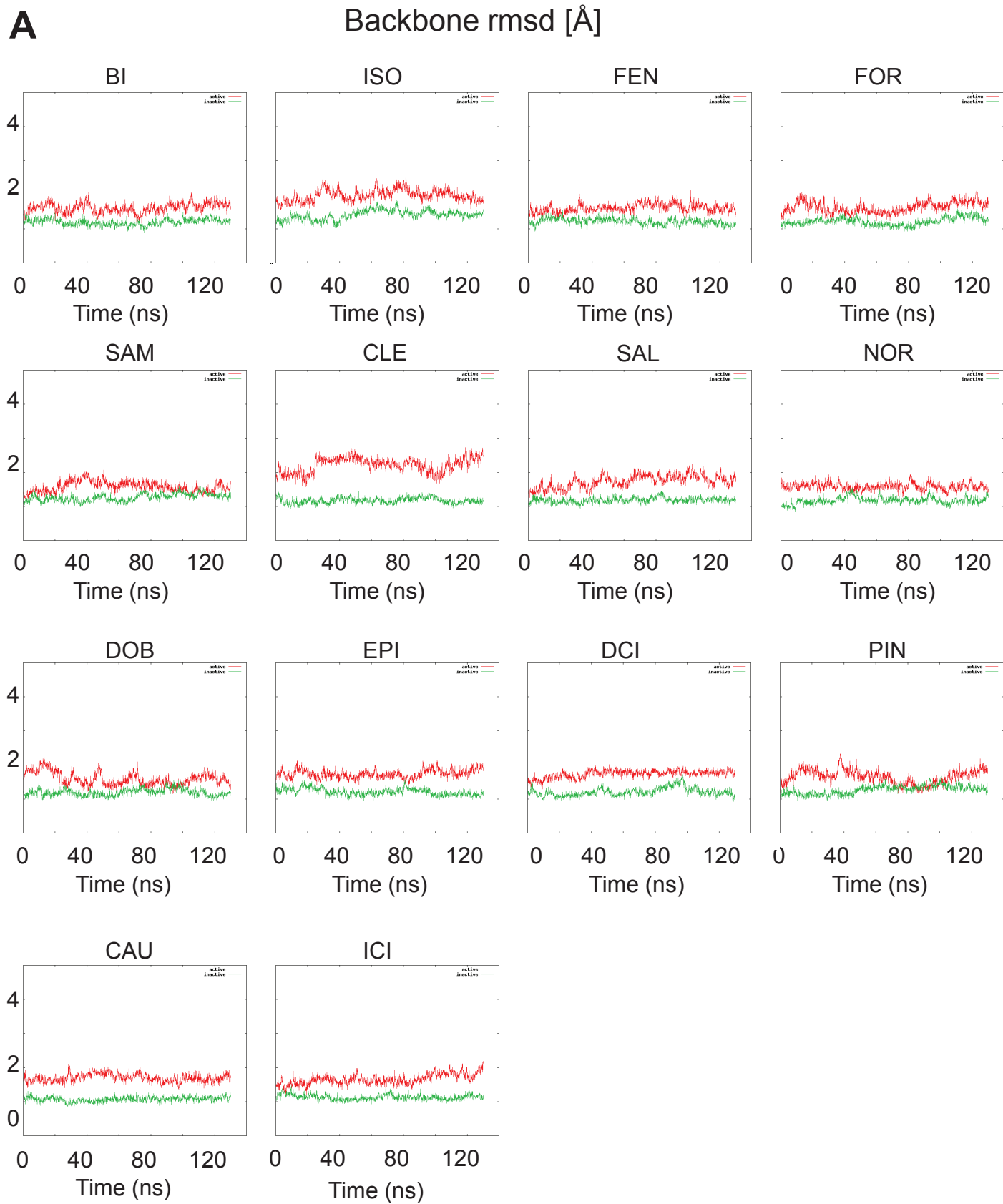

**B**Helix6-Helix3 distance [ $\text{\AA}$ ]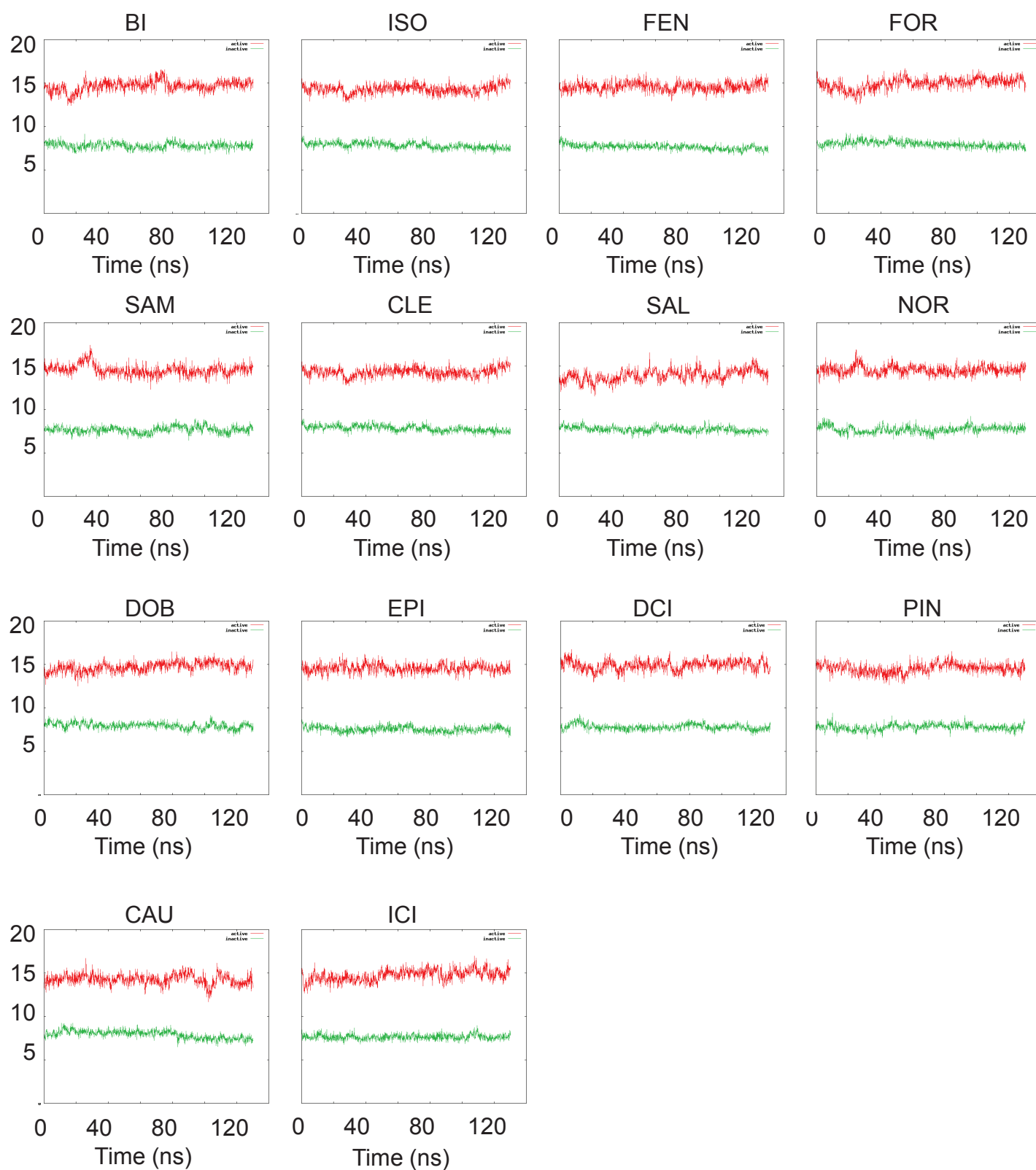

Supplement: S4 Fig — The results of G-protein-active R* state and inactive R state are colored red and green, respectively. (A) Backbone RMSD of all region relative to the initial crystal structure (PDB ID: 2RH1, 3SN6). (B) Helix 3–helix 6 distance, which is monitored by Arg1313.50–Leu2726.34 Cα atom distance. (PDF) [file pone.0155816.s004.pdf]

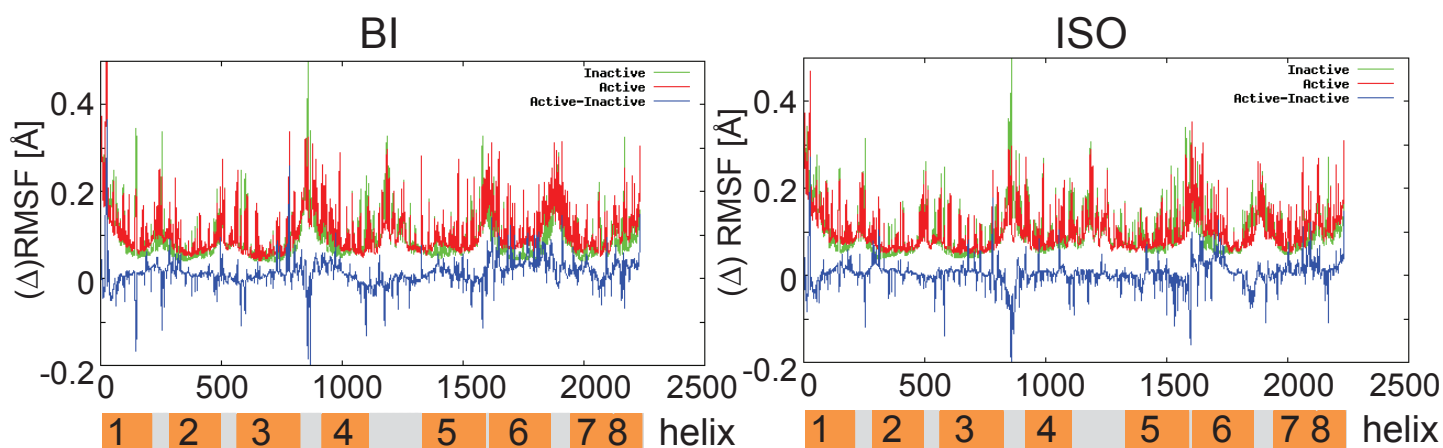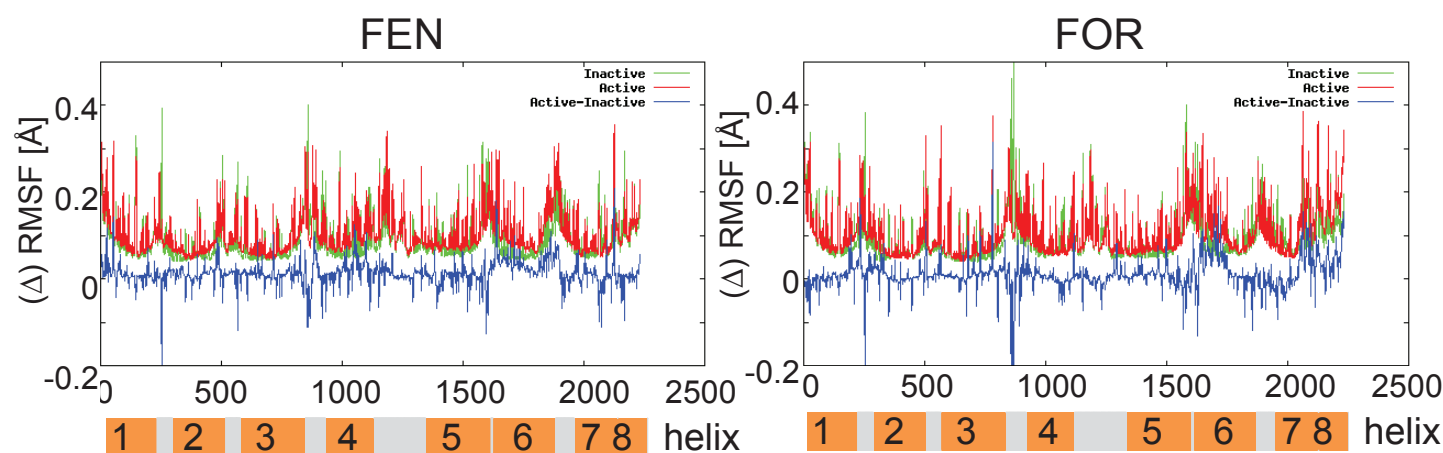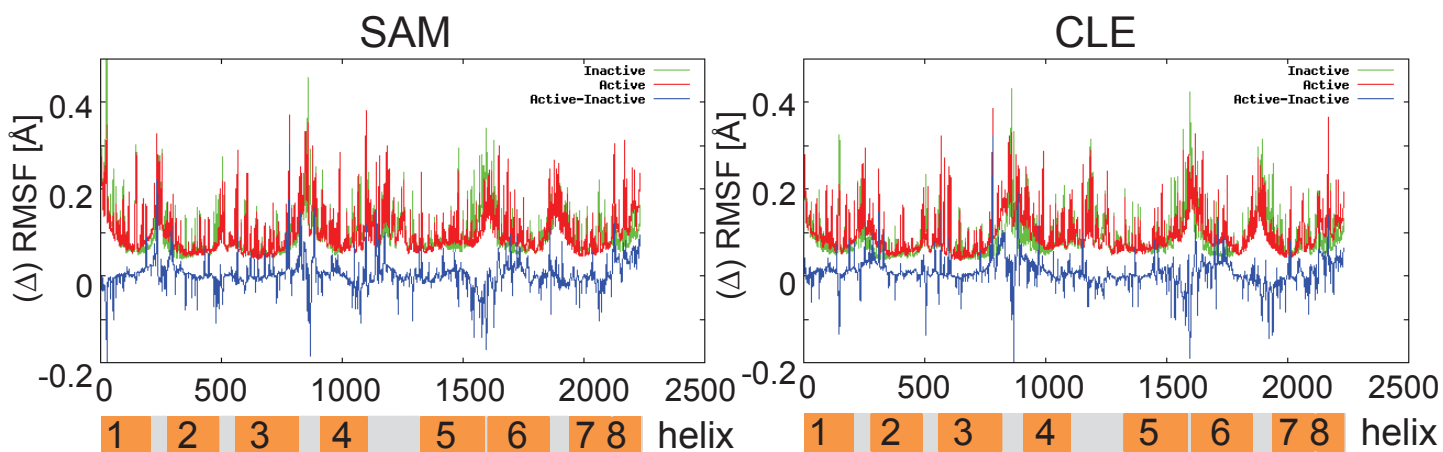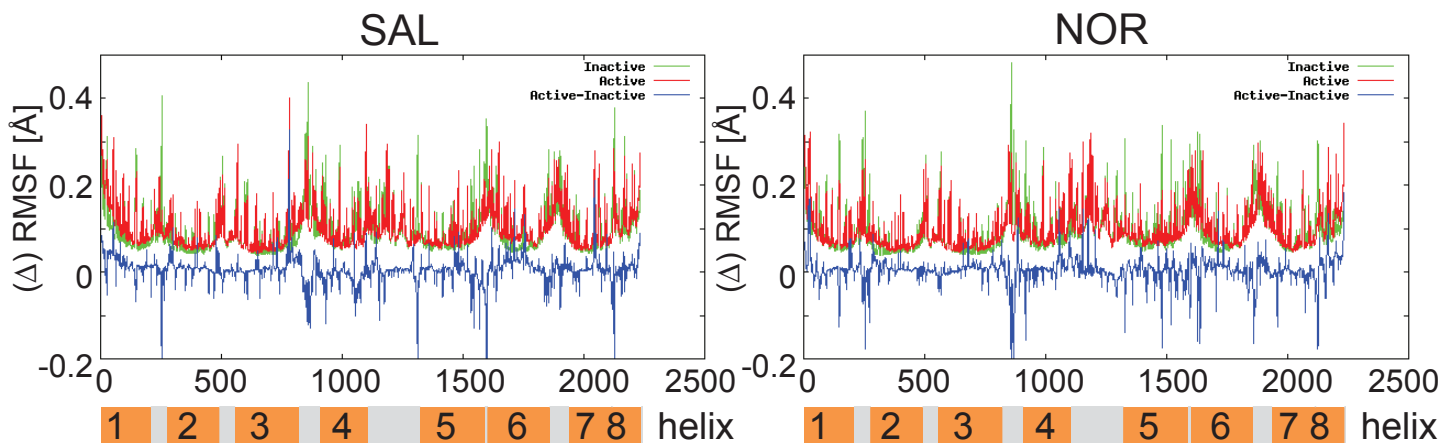

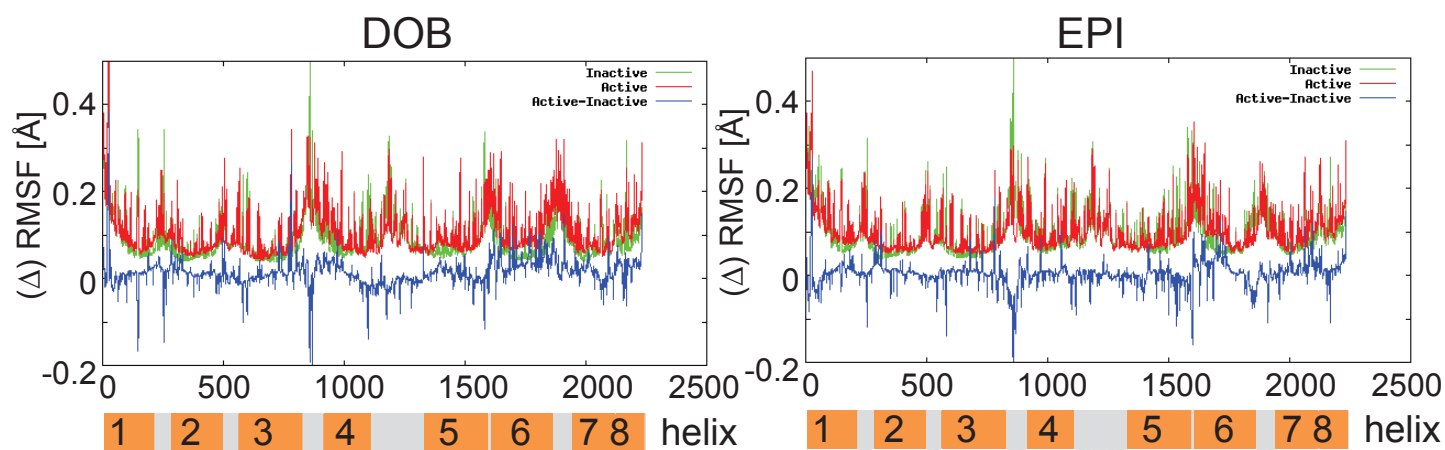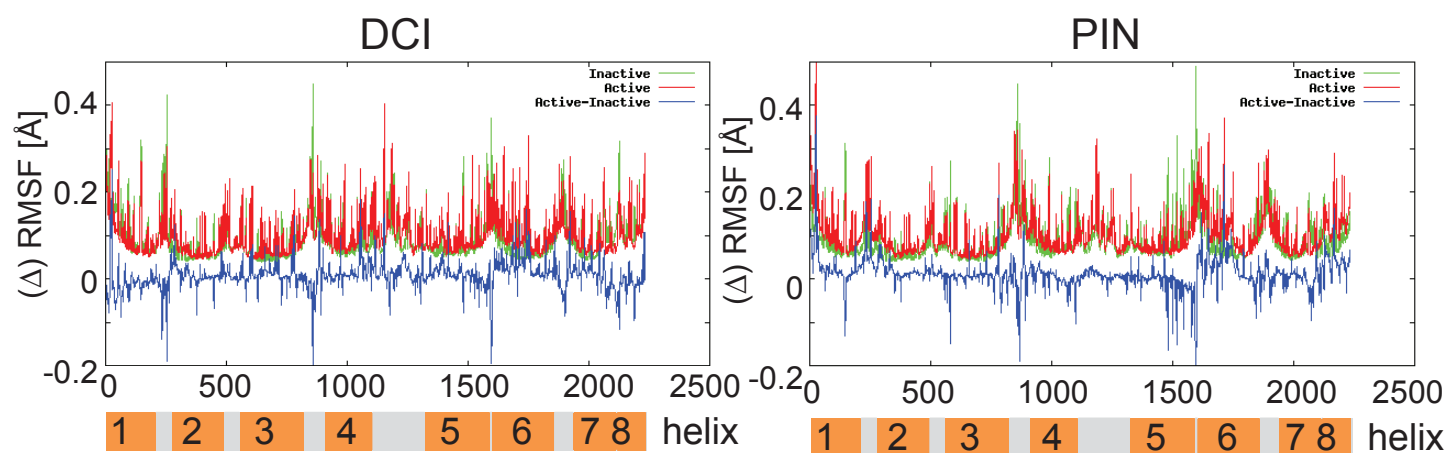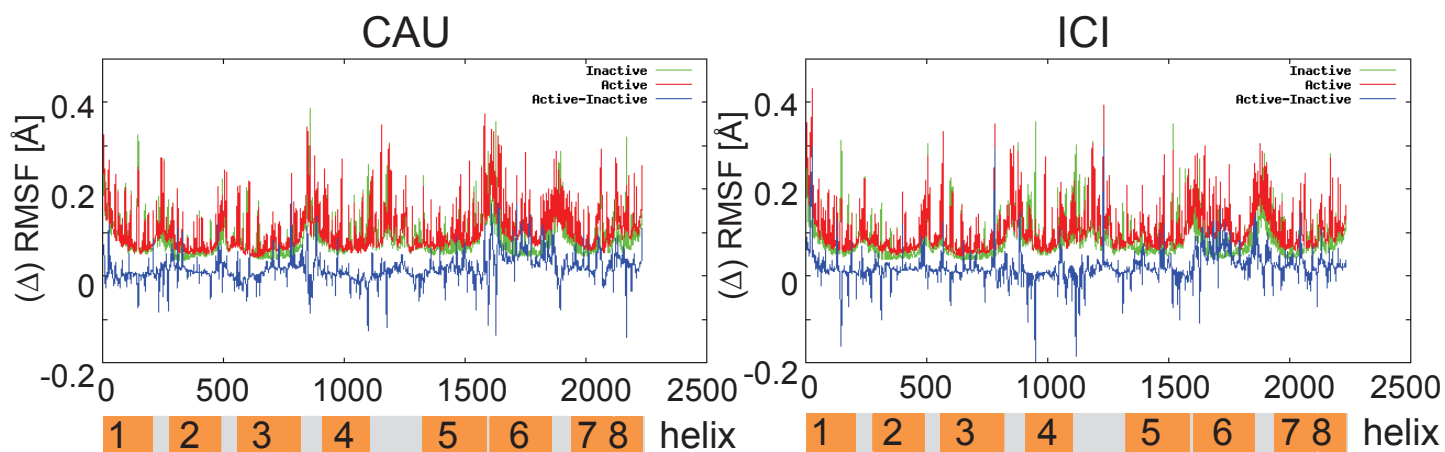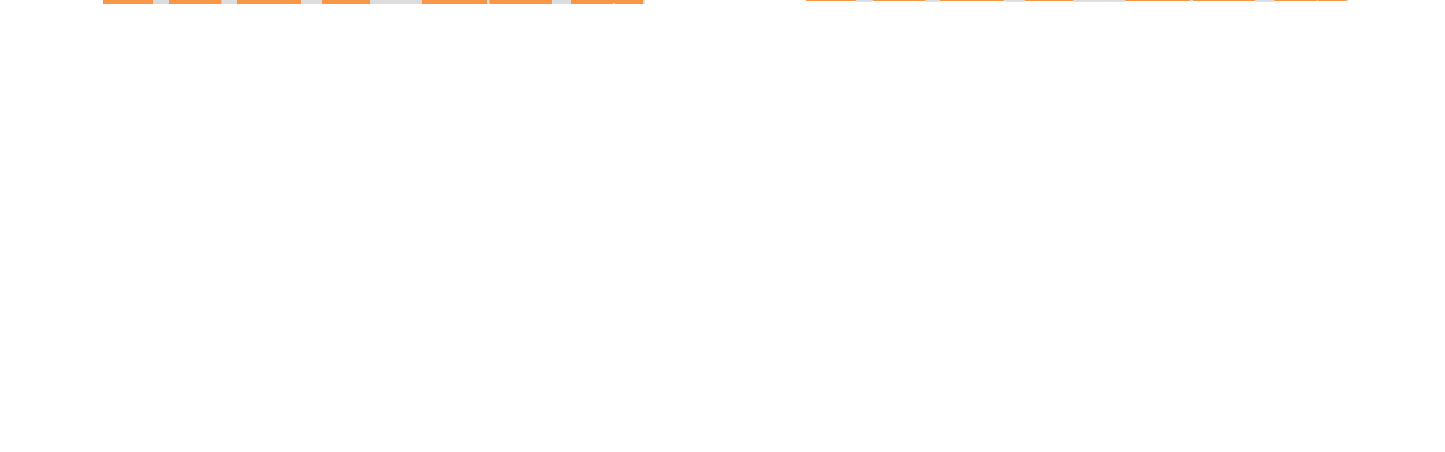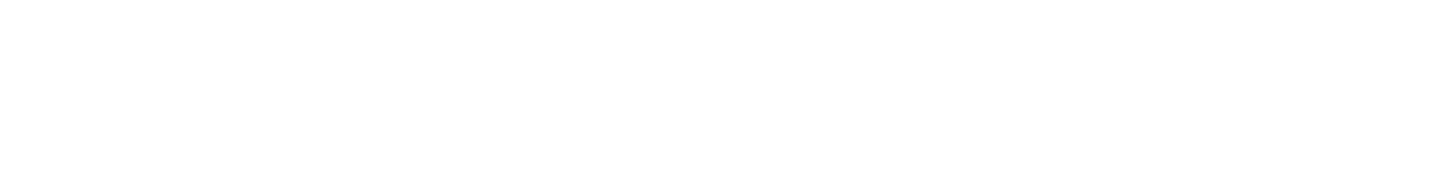

Supplement: S5 Fig — The RMSF of G-protein-active R* state and inactive R state and ΔRMSF are colored red, green and blue, respectively. The region of helices are shown as orange bar. (PDF) [file pone.0155816.s005.pdf]

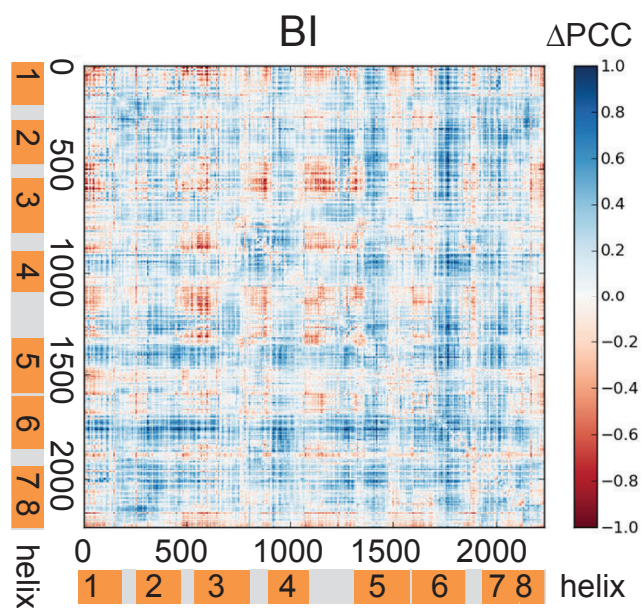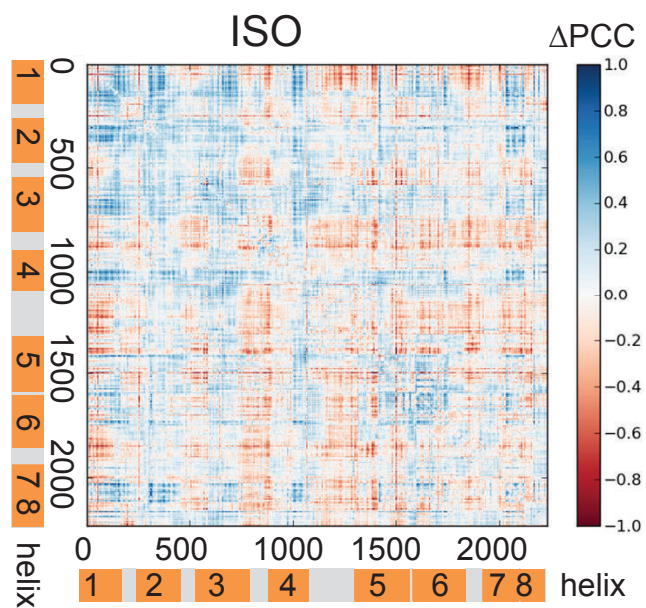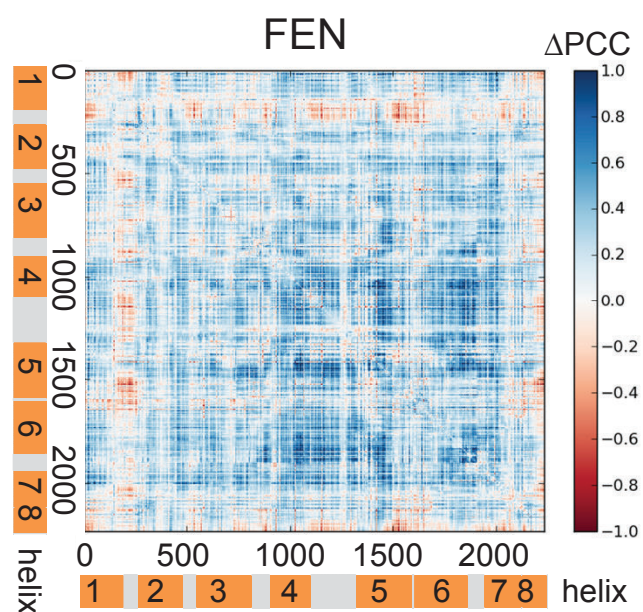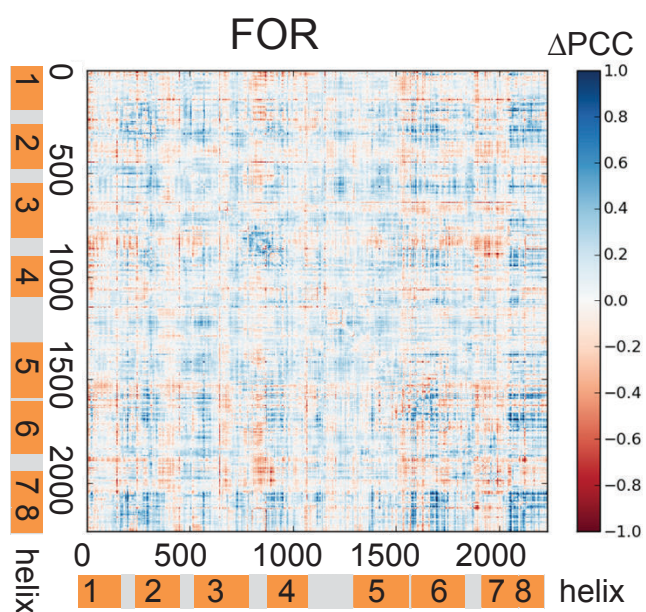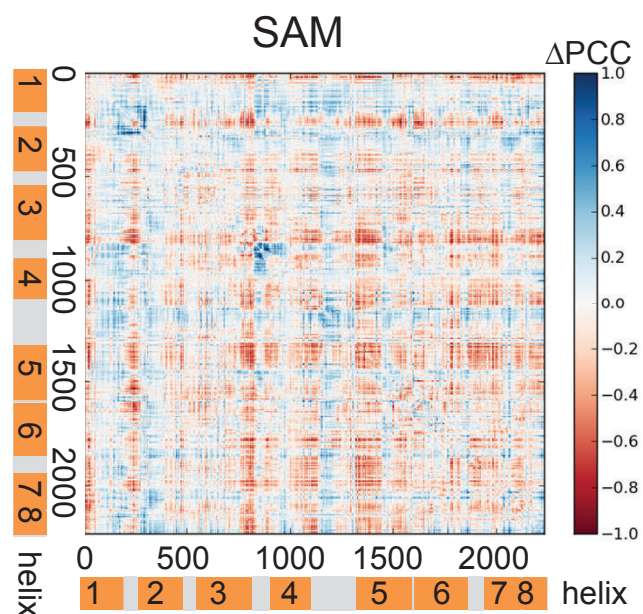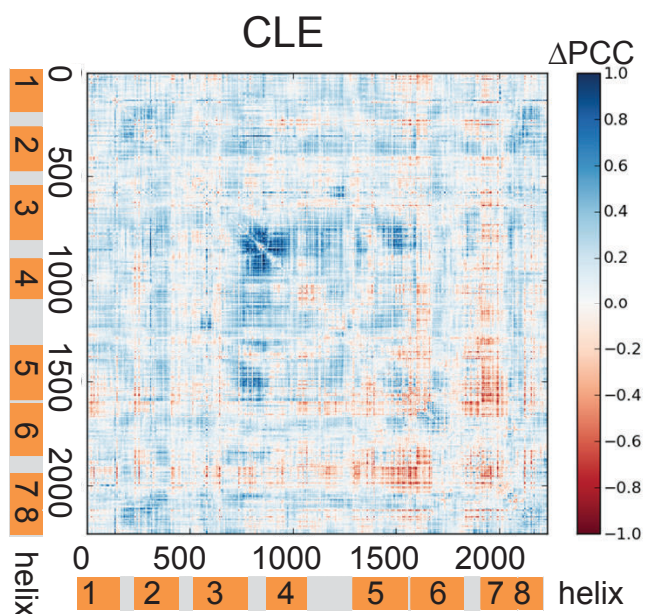

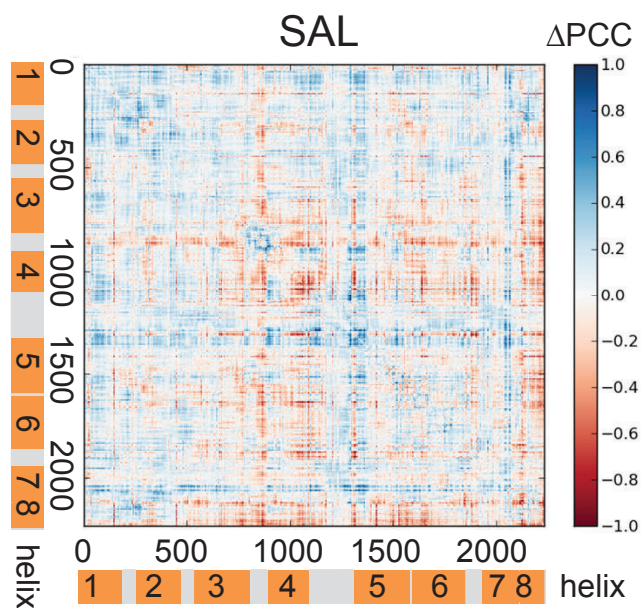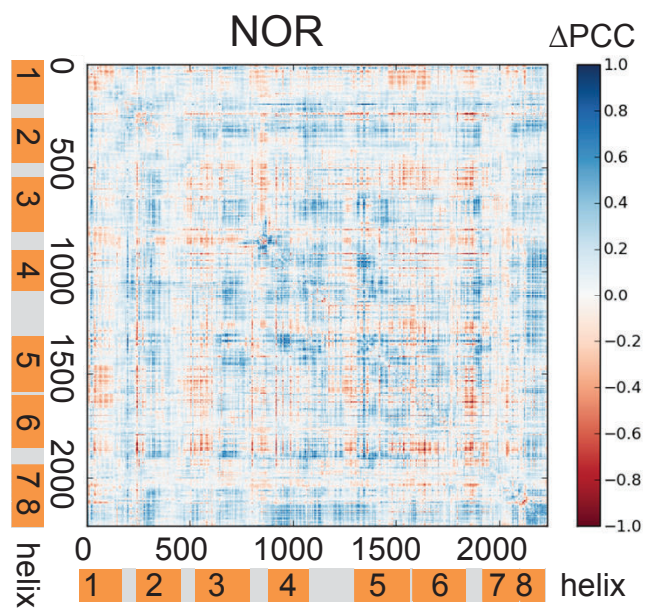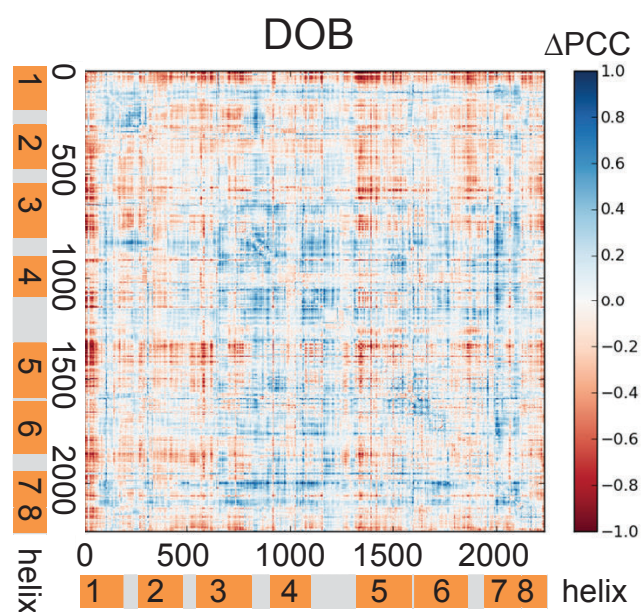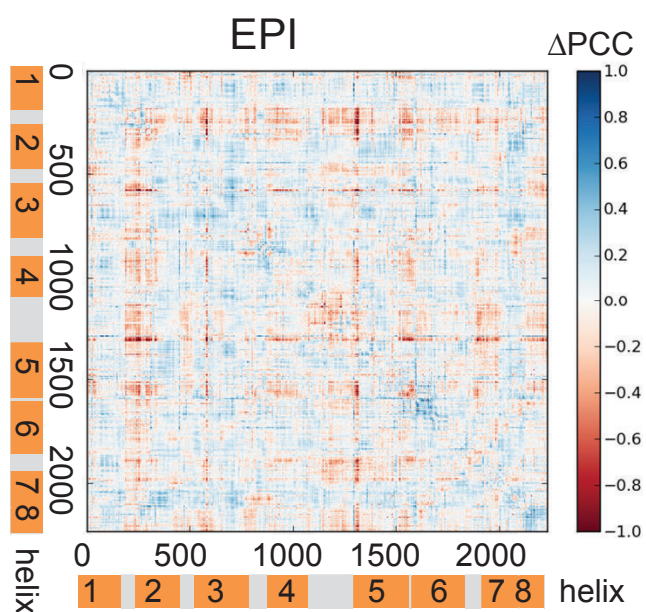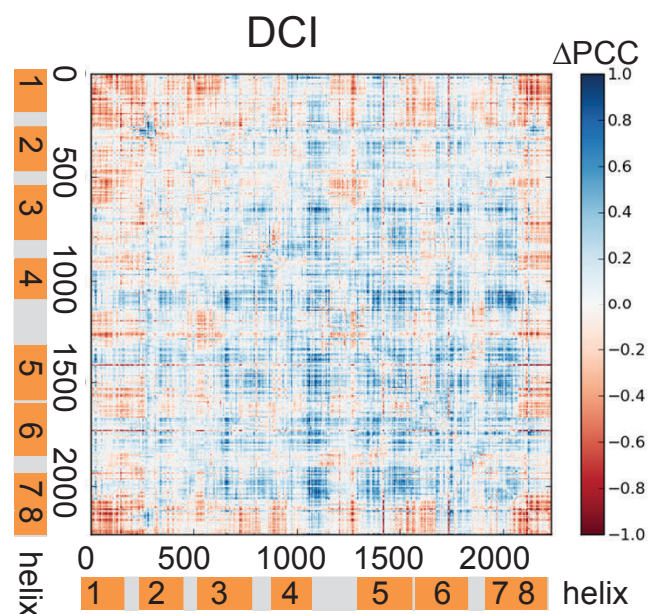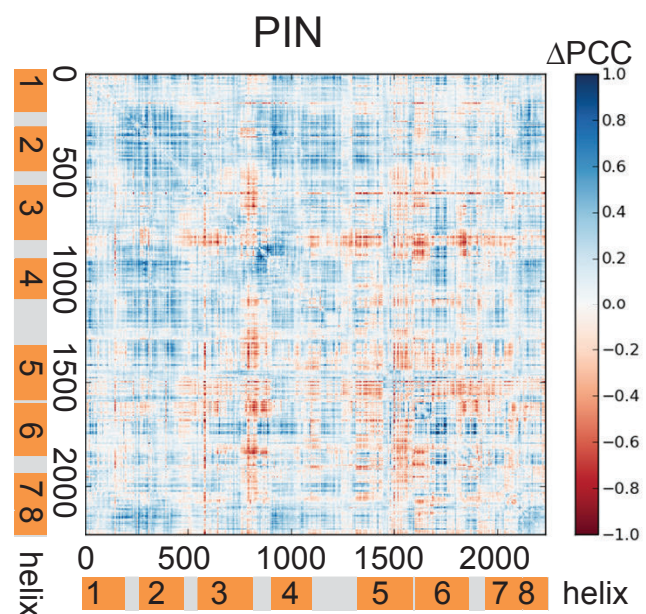

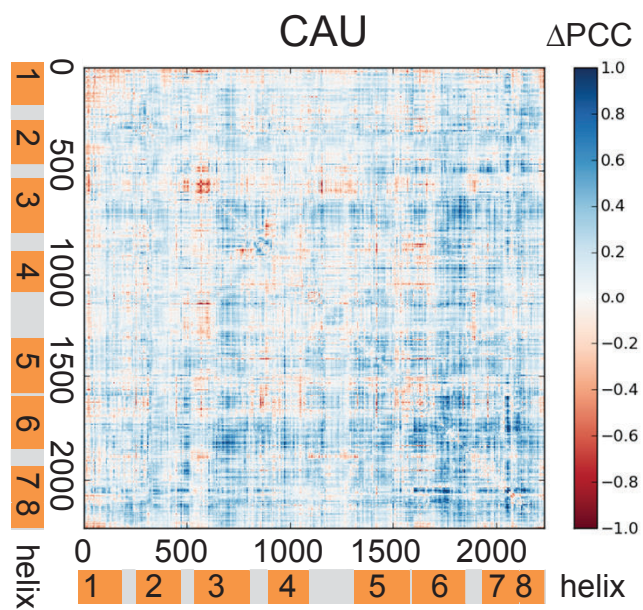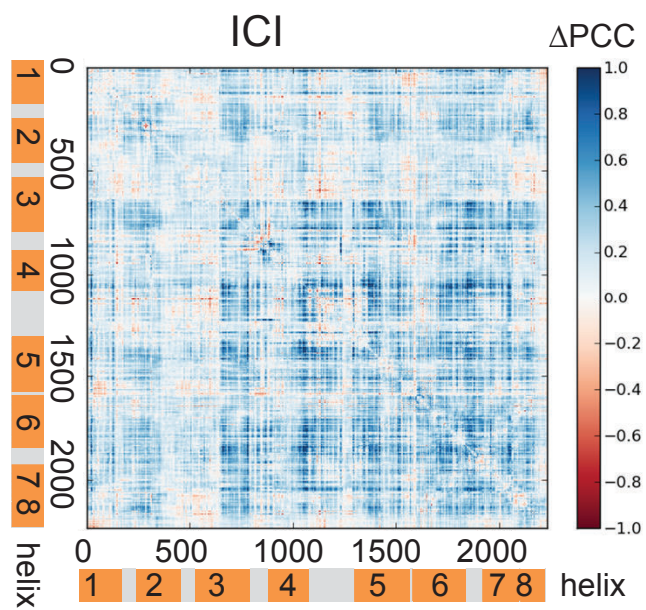

Supplement: S6 Fig — The positive value and negative values are colored blue and red, respectively. The region of helices are shown as orange bar. (PDF) [file pone.0155816.s006.pdf]

**A**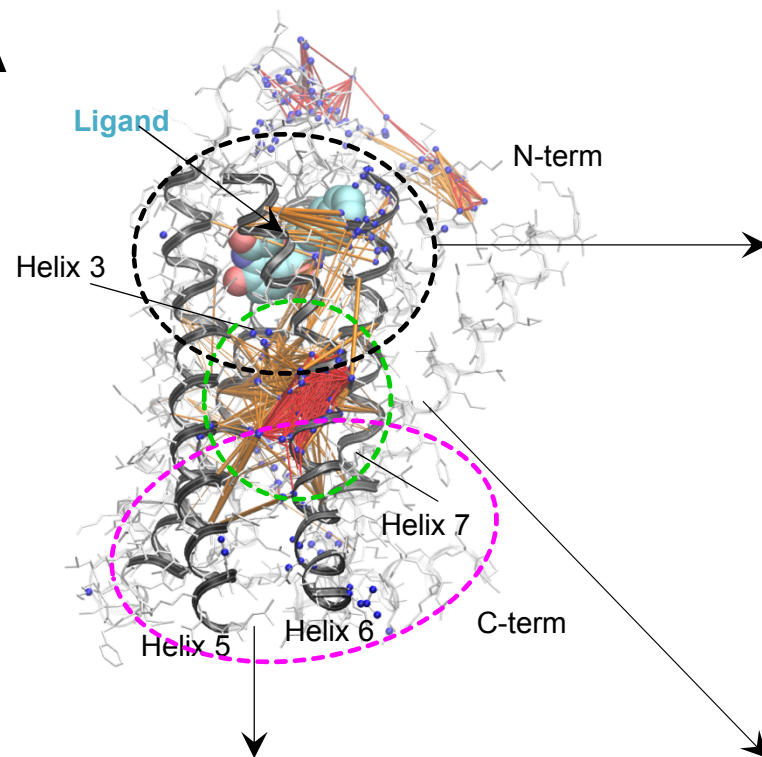**B** Ligand-binding site (side view)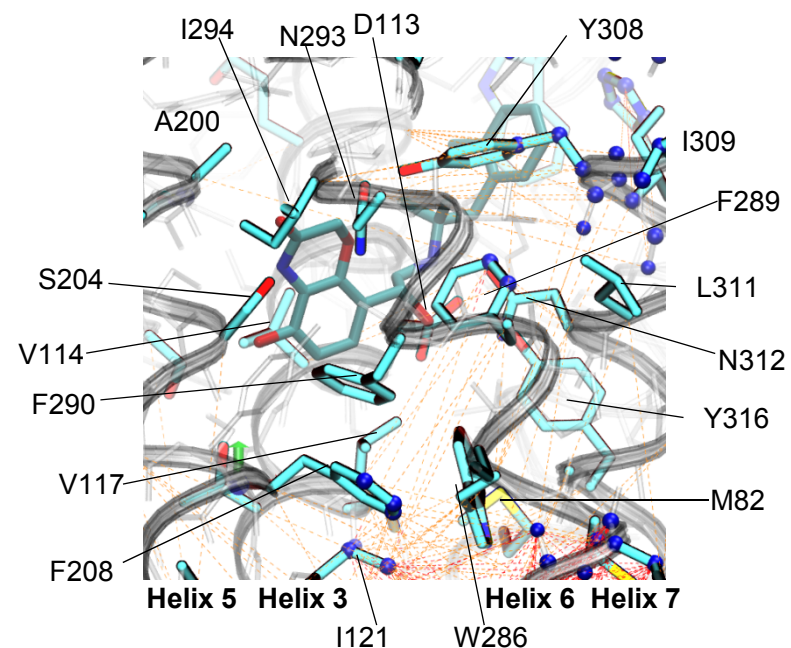**C** G-protein-binding site (bottom view)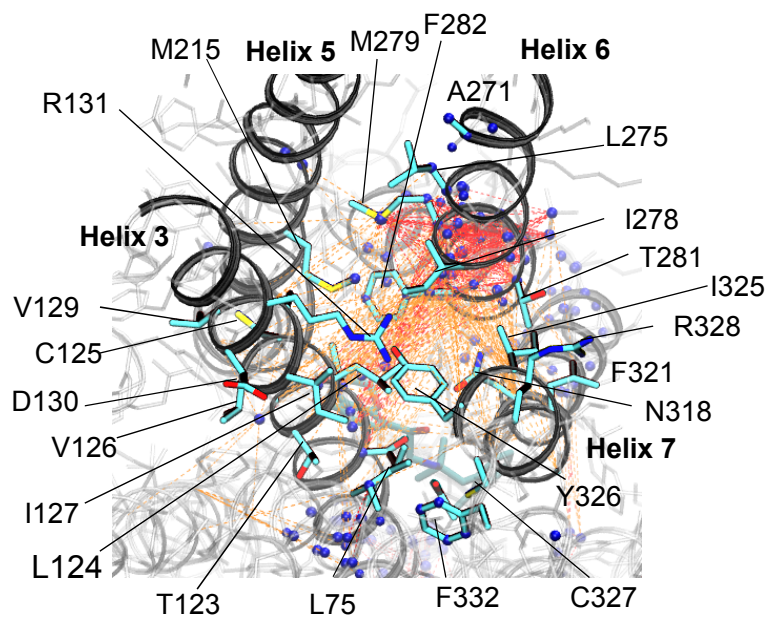**D** Connector (side view)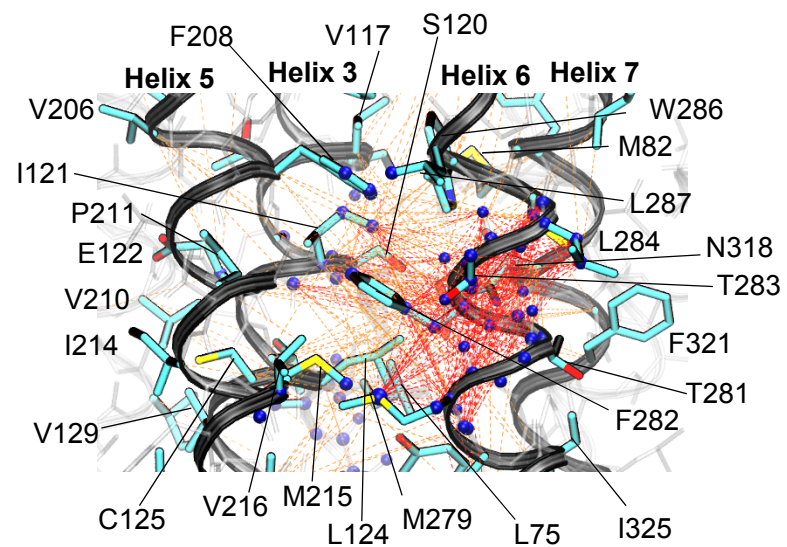

Supplement: S7 Fig — The residues that constitute the G-protein-linked fluctuating network are shown by cyan stick model. The fluctuating atomsG-protein are shown by blue spheres. The atom–atom couplingsG-protein are shown by red or orange dashed line with the same coloring as Fig 3B. (A) Overall view of the G-protein-linked fluctuating network in the same position as right view of Fig 3B. (B) Side view of the ligand-binding site. (C) Bottom view from the intracellular region of the G-protein-binding site. (D) Side view of the connector region. (PDF) [file pone.0155816.s007.pdf]

**A**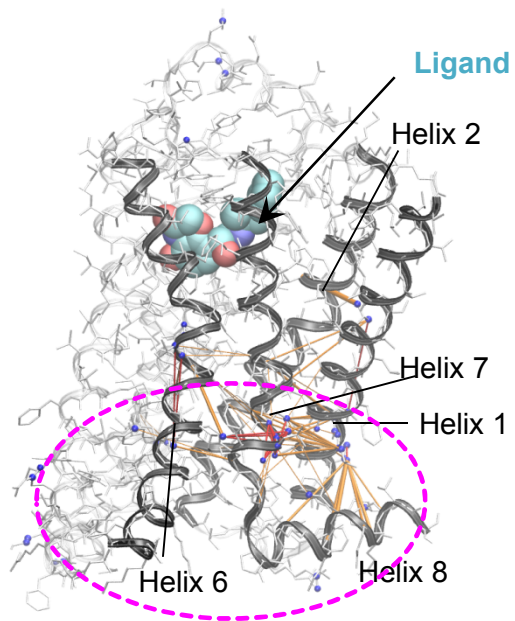**B**

Side view

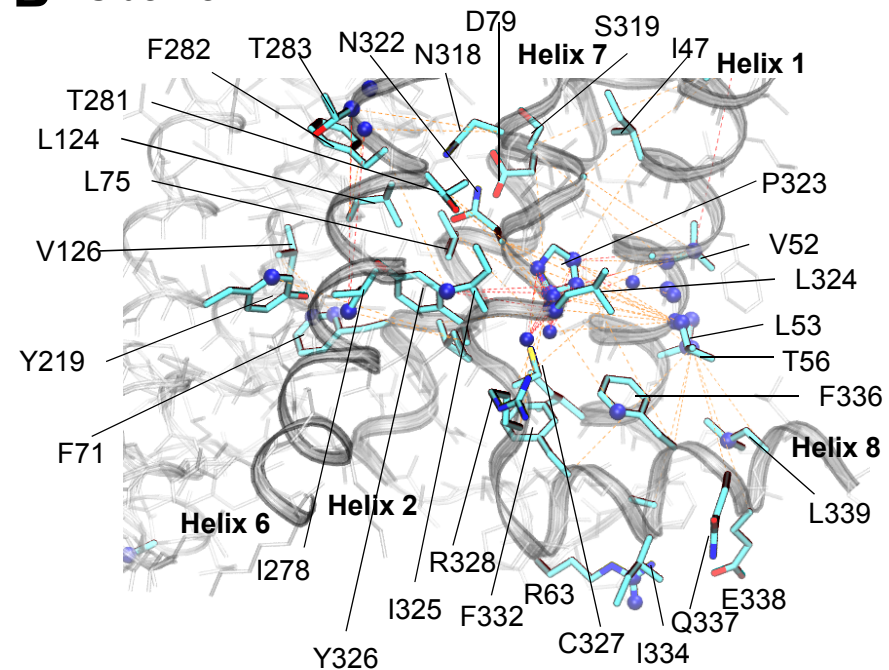**C**

Bottom view

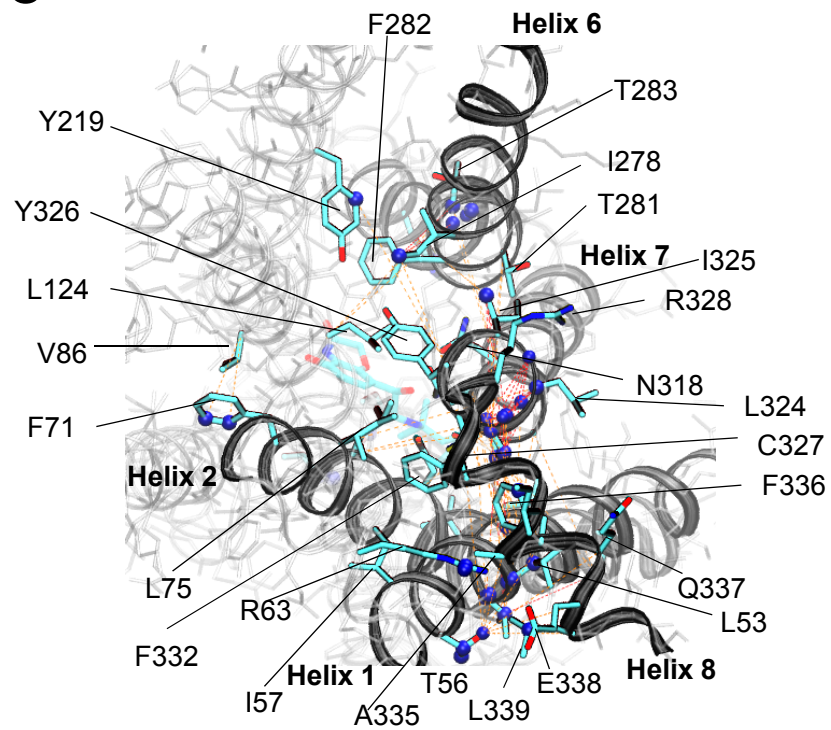

Supplement: S8 Fig — The residues that constitute the β-arrestin-linked fluctuating network are shown by cyan stick model. The fluctuating atomsβ-arrestin are shown by blue spheres. The atom–atom couplingsβ-arrestin are shown by red or orange dashed line with the same coloring as Fig 4B. (A) Overall view of the β-arrestin-linked fluctuating network in the same position as left view of Fig 4B. The β-arrestin-binding site are shown by dashed magenta ellipse. (B) Side view of the β-arrestin-binding site. (C) Bottom view from the intracellular region of the β-arrestin-binding site in the same position as S7C Fig. (PDF) [file pone.0155816.s008.pdf]
