# Supplementary material for: G-Protein/β-Arrestin-Linked Fluctuating Network of G-Protein-Coupled Receptors for Predicting Drug Efficacy and Bias Using Short-Term Molecular Dynamics Simulation
Source: PLoS One. 2016 May 17;11(5):e0155816. doi: 10.1371/journal.pone.0155816 (PMC4871340; doi:10.1371/journal.pone.0155816)
Supplement: S1 Text — (DOC) [file pone.0155816.s013.doc]

# **S1 Text. Supplementary discussion**

The G-protein-linked fluctuating network demonstrates tight connections between the ligand-binding site, the connector region, and the G-protein-binding site. For the ligand-binding site, the G-protein-linked fluctuating network includes helix 5 residues, such as Val206^5.45^ and Phe208^5.47^, and helix 5 couples with the connector region, including helix 3, Ser120^3.39^, Ile121^3.40^, and Glu122^3.41^; helix 6, Met279^6.41^, Thr283^6.43^, and Leu284^6.46^. This is consistent with previous studies indicating helix 5 movement was the primary change upon binding [1]. In addition, the G-protein-linked fluctuating network involves not only helix 5 residues but also more than half of the ligand-contact residues [2], ranging broad area of ligand-binding site, for example, Phe289^6.51^, which interacts with aromatic ring of ligand by a π–π interaction, Val114^3.33^, Val117^3.36^, Tyr308^7.35^, and Ile309^7.36^, which have van der Waals contact with a ligand, Asn312^7.39^, which forms a hydrogen bond with a ligand. These ligand-contact residues in helices 3, 6, and 7 also couples with the connector region, including helix 3, Ser120^3.39^, Ile121^3.40^, and Glu122^3.41^; helix 6, Phe282^6.44^ and Thr283^6.45^, directly or through Trp286^6.48^ and/or Phe289^6.51^. This result shows that the fluctuating network is controlled not only by single hydrogen bond between helix 5 and a ligand but also by comprehensive interaction of multiple residues, which suggests that these multiple residues induces the G-protein signaling as an integrated manner. Between the ligand-binding and G-protein-binding sites, there is a tightly coupled “core” of the G-protein-linked fluctuating network formed by helix 3, Ser120^3.39^, Ile121^3.40^, and Glu122^3.41^; helix 6, Ile278^6.40^, Met279^6.41^, Gly280^6.42^, Thr281^6.43^, Phe282^6.44^, Thr283^6.45^, and Leu284^6.46^; helix 5, Phe208^5.47^ and Met215^5.54^. This core has coupling not only with above-mentioned ligand-binding site but also with broad region of helix 7 residues, Tyr316^7.43^, Asn318^7.45^–Phe321^7.48^; helix 3, Thr123^3.42^–Val129^3.48^; helix 5, Pro211^5.50^, Ile214^5.53^, and Val216^5.55^. Furthermore, the core residues also connect with upper part of the G-protein-binding site, including Ile325^7.52^ and Tyr326^7.56^ in NPxxY motif and Asp130^3.49^ and Arg131^3.50^ in DRY motif, both of which are key region for GPCR activation [3,4]. Thus, the tightly coupled fluctuation in helices 3, 5, 6, and 7 demonstrates how the large conformational changes of Ile121^3.40^ and Phe282^6.44^ are induced from the ligand-binding site and transfer the signal to the G-protein-binding site. In the G-protein-binding region, Tyr219^5.58^ and Tyr326^7.53^ are characteristic residues that define the receptor state [1]. In this study, Tyr326^7.53^ was included in the G-protein-linked fluctuating network. Tyr219^5.58^ itself is not in the network, but all of its contacting residues (Ala128^3.47^, Met215^5.54^, Ile278^6.40^, and Met279^6.41^) are included in the network. Furthermore, the G-protein-linked fluctuating network involves a part of G-protein-contact residues [2], Arg131^3.50^, Ala271^6.33^, and Leu275^6.37^.

In addition to these integrated network in helices 3, 5. 6, and 7, we identified additional network in ECL1 and ECL2. These loops overlap with the allosteric ligand-binding site of other aminergic GPCR [5,6], which suggest that these loops of β*_2_*AR might be an allosteric site and affect conformational transition through the atom-atom couplings with adjacent helix 7.

Reference

1. Dror RO, Arlow DH, Maragakis P, Mildorf TJ, Pan AC, Xu H, et al. Activation mechanism of the β2 -adrenergic receptor. Proc Natl Acad Sci USA. 2011;108: 18684–18689. doi:10.1073/pnas.1110499108

2. Venkatakrishnan AJ, Deupi X, Lebon G, Tate CG, Schertler GF, Babu MM. Molecular signatures of G-protein-coupled receptors. Nature. Nature Publishing Group; 2013;494: 185–194. doi:10.1038/nature11896

3. Rosenbaum DM, Rasmussen SGF, Kobilka BK. The structure and function of G-protein-coupled receptors. Nature. 2009;459: 356–363. doi:10.1038/nature08144

4. Angel TE, Chance MR, Palczewski K. Conserved waters mediate structural and functional activation of family A (rhodopsin-like) G protein-coupled receptors. Proc Natl Acad Sci U S A. 2009;106: 8555–8560. doi:10.1073/pnas.0903545106

5. Dror RO, Green HF, Valant C, Borhani DW, Valcourt JR, Pan AC, et al. G-protein-coupled receptor by allosteric drugs. Nature. 2013;503: 295–299. doi:10.1038/nature12595

6. Jeffrey Conn P, Christopoulos A, Lindsley CW. Allosteric modulators of GPCRs: a novel approach for the treatment of CNS disorders. Nat Rev Drug Discov. 2009;8: 41–54. doi:10.1038/nrd2760
